# Barriers and facilitators to healthcare utilization amongst people living with sickle cell disease in the United States: A scoping review

**DOI:** 10.1371/journal.pone.0349441

**Published:** 2026-07-06

**Authors:** Christina Ruan, Joyce Gyamfi, Nana Osei-Tutu, Shreya Meda, Lydia Samuels, Nousheen Inayat, Sukruthi Thunga, Elizabeth Noble, Dorice L. Viera, Deborah Adenikinju, Charmaine Royal, Angela Odoms-Young, Prince Michael Amegbor, Emmanuel Peprah

**Affiliations:** 1 Department of Global and Environmental Health, NYU School of Global Public Health, New York, New York, United States of America; 2 Georgetown University Medical Center, Washington, District of Columbia, United States of America; 3 NYU Grossman School of Medicine, NYU Health Sciences Library, New York, New York, United States of America; 4 Department of African and African American Studies, Duke University, Durham, North Carolina, United States of America; 5 College of Human Ecology, Division of Nutritional Sciences, Cornell Center for Health Equity, Cornell University, Ithaca, New York, United States of America; PLOS: Public Library of Science, UNITED KINGDOM OF GREAT BRITAIN AND NORTHERN IRELAND

## Abstract

**Background:**

Sickle cell disease (SCD) stands as one of the most prevalent genetic disorders in the United States (U.S.) that causes severe consequences such as organ damage and excruciating pain. Alarmingly, recent literature indicates a decline in the number of people living with SCD (PLWSCD) seeking professional care – hinting at an avoidance of the healthcare system. Therefore, this scoping review synthesizes the evidence regarding barriers and facilitators influencing healthcare utilization among PLWSCD within the U.S.

**Methods:**

To map the current literature on SCD management and provide a comprehensive overview of the current knowledge gaps regarding healthcare utilization for PLWSCD, a scoping review was conducted. A systematic search of articles reporting on the utilization of healthcare among PLWSCD in the U.S using seven data sources was conducted on March 24, 2023, without any restrictions on publication date and language. To capture any additional articles, the search was updated on March 4, 2024. Two reviewers independently assessed studies for inclusion, data extraction, and risk of bias (RoB).

**Main results:**

A total of 708 articles were screened; 70 met the study criteria. Results indicated that the four most common barriers were social (*n* = 25) interpersonal (*n* = 23), economic (*n* = 15), and institutional level factors (*n* = 11). The top four most common facilitators were technology (*n* = 9), education (*n* = 7), autonomy (*n* = 6), and a positive patient-provider relationship (*n* = 6). The most common forms of healthcare utilization were inpatient or hospital admissions (*n* = 19) and emergency department (ED) visits (*n* = 18). Evidence-based interventions (EBI) found to decrease healthcare avoidance included individualized pain plans (IPPs) (*n* = 4) and quality improvement (QI) strategies (*n* = 3).

**Conclusion:**

This scoping review identified complex multilevel barriers that impede healthcare utilization, and facilitators likely to promote healthcare utilization among PLWSCD in the U.S. Future research should prioritize developing and evaluating comprehensive, multi-level interventions that address identified barriers while leveraging facilitators to improve healthcare engagement and outcomes for this vulnerable population. Healthcare systems and health policies must urgently adopt and integrate evidence-based strategies to rebuild trust and ensure equitable, accessible care for PLWSCD.

## Introduction

Sickle cell disease (SCD) is a genetic blood disorder that arises from a point mutation in the β-globin gene (HBB), resulting in glutamate (E/Glu) being substituted by valine (V/Val) [1]. Hemoglobin with this mutation is referred to as HbS, as opposed to the normal adult HbA [1]. Under normal oxygen concentrations, the mutation is benign; however, under low oxygen concentrations, HbS polymerizes and forms fibrous precipitates that deform the biconcave shape of the erythrocytes to a crescent shape [1]. These repeated episodes of polymerization decrease the cell membrane’s elasticity, causing damage and premature rupture of erythrocytes and anemia [1]. Furthermore, the occlusion of blood vessels from the crescent-shaped red blood cells results in extreme pain and vaso-occlusive crises (VOC) with multiorgan-level damage and increased mortality [2]. Other common symptoms consist of anemia, strokes, dactylitis, jaundice, and acute chest syndrome. Complications include leg ulcers, early-onset gallstones, splenic sequestration, retinopathy, and nephropathy [2]. In the United States (U.S.), diagnosis typically occurs through newborn screening in which a combination of biochemical and molecular tests is used as confirmation markers [1]. Treatment strategies for SCD are inherently multifaceted, reflecting the complexity and chronic nature of the condition. Pharmacological management plays a central role in this therapeutic landscape, as patients are frequently prescribed multiple medications to alleviate pain and further management complications. On average, individuals with SCD are prescribed approximately 4.5 medications per prescription [3] highlighting the prevalence of polypharmacy. As polypharmacy is often associated with multimorbidity [3], this relationship underscores the necessity of multidisciplinary care to optimize treatment outcomes and minimize the risks of drug interactions, toxicity, and adverse effects – especially for individuals who present with co-existing health conditions in conjunction to SCD. Among the most prescribed therapies are vitamins– particularly folic acid to support red blood cell production – antianemia agents such as L-glutamine and antineoplastic drugs such as hydroxyurea (HU) to reduce the frequency of VOCs and the need for blood transfusions [3]. Recent years have witnessed promising advancements in SCD management. Two additional cell-based gene therapies – Casgevy and Lyfgenia – were approved by the FDA in December 2023, offering renewed hope for PLWSCD navigating the complexities of this relentless condition [4]. These promises fall short due to their financial inaccessibility with Casgevy sitting at a price point of $2.2 million and Lyfgenia at $3.1 million [4].

The management of SCD requires adherence to rigorous practice guidelines which encompasses continuous monitoring, frequent appointments over a few months, obtaining prescribed medications, and adhering to routine immunizations – all to forestall the onset of further complications [5]. Over a lifetime, the costs of treating SCD exceeds over $1.6 million per person [4]. For individuals with commercial health insurance, out of pocket costs can be more than $40,000 [4] – a figure that is nearly three times more than individuals without SCD. These costs fail to quantify the indirect costs of absenteeism, presenteeism, and personal setbacks attributed to SCD. Individuals affected by this condition also face a staggering reality – despite advancements in pharmacological treatments, their average life expectancy in the U.S. falls short by approximately 20 years when compared to the general population [6]. These pharmaceutical costs also fail to quantify the indirect costs of SCD from the years of lost life and mental health challenges experienced by affected individuals and loved ones.

The dynamics of SCD-related healthcare utilization are complex due to various influencing factors. The decision to seek healthcare hinges on several key elements: the severity of illness or disability, individuals’ awareness of their healthcare needs and desires, and the accessibility of healthcare services [7]. Effective pain management is crucial in the comprehensive care of individuals with SCD as its quality is closely tied to an individual’s access to healthcare services, including the range of services available and their ability to navigate these resources. Chronic pain is the clinical determinant of quality of life for PLWSCD [8]. Consequently, many seek care for their pain crises in emergency departments (ED), which can result in lengthy hospitalizations. Notwithstanding in many cases, pain resolution remains elusive prior to discharge [9]. A lack of effective pain management strategies results in many PLWSCD opting for at-home management during VOC; suggesting a pervasive phenomenon of healthcare system avoidance [9].

Sickle cell anemia (SCA) – an autosomal recessive genetic disorder – is the most severe form of SCD [10]. Symptoms include extreme fatigue, frequent infections due to a suppressed immune system, onset of delayed puberty, and visual impairments [10]. Life threatening complications encompasses stroke, pulmonary hypertension, thrombosis, and blindness [10]. As SCA is the most prevailing cause of childhood stroke in the U.S, recommendations developed by the National Institute of Health’s National Heart, Lung, and Blood Institute included preventative care efforts such as stroke screenings and medications such as HU to reduce painful episodes [9]. A study conducted in 2019 by the Centers for Disease Control and Prevention (CDC) found that less than half of the affected children and adolescents received the annual stroke screening as well as the recommended medication for SCD management [9]. The decision to endure excruciating pain at home rather than seek medical intervention at healthcare facilities speaks volumes about the barriers that cast a shadow over the healthcare landscape for PLWSCD. The underlying factors contributing to this phenomenon are multifaceted and deeply rooted in the systemic intricacies of healthcare delivery – factors that are exacerbated with individuals diagnosed with SCA as the disorder predominantly affects people of African descent [6]. These barriers obstruct equitable access to care and essential pain management interventions. Such divergences underscore the importance of addressing systemic gaps that impede equitable access to care and essential pain management resources.

Moreover, the transition from pediatric to adult-focused healthcare settings unveils another layer of complexity, one fraught with heightened levels of vulnerability and, tragically, elevated mortality rates [2]. This transitional period serves as a stark reminder that barriers to care may manifest at various stages of an individual’s healthcare journey; some obstacles are transient, while others persist and evolve. As such, a nuanced understanding of these multifaceted barriers is essential to effect meaningful change and ensure equitable access to good quality healthcare for all individuals affected by SCD, regardless of age or circumstance.

To address the gaps in the literature, the aim of this scoping review is to identify multi- level factors linked to the utilization of healthcare services among PLWSCD in the U.S. Fostering a landscape where care is readily accessible for optimal management of SCD and improvements in quality of life requires the deconstruction of barriers. A comprehensive understanding of the barriers and facilitators to healthcare utilization has the potential to inform the development of targeted interventions. The questions raised in this scoping review are 1) What are the specific barriers that limit optimal management of SCD care? 2) What facilitators can policymakers and external stakeholders implement to address the troubling trend of healthcare avoidance?

## Methods

### Materials and methods

This study is registered in Open Science Framework (OSF), an open-source cloud-based project management platform [https://doi.org/10.17605/OSF.IO/KUAZW]. This scoping review was guided by a consolidated checklist of the Arksey O’Malley framework and the Preferred Reporting Items for Systematic Reviews and Meta-Analyses extension for Scoping Reviews (PRISMA-ScR) Checklist (**S1 File**) to ensure a comprehensive overall of articles [11,12]

### Inclusion and exclusion criteria

The inclusion criteria encompassed studies meeting the following specifications: (1) focused on individuals diagnosed with SCD, (2) conducted in the U.S., (3) addressed any aspect of SCD perception (4) examined healthcare disparities, barriers, utilization, and/or interventions. Studies were excluded if they failed to meet any of the following criteria or were designated as a protocol, commentary, or systematic review. Language and publication year restrictions were not imposed, ensuring a comprehensive scope of literature review.

The stringent inclusion and exclusion criteria were applied to maintain the relevance and rigor of the study selection process and to focus on SCD studies reporting primary data from PLWSCD.

### Information sources

The following seven databases were searched: PubMed/Medline (Ovid), Embase, the Cochrane Library, the Cumulative Index to Nursing and Allied Health (CINAHL), Global Health (Ovid), APA PsycInfo, and Web of Science.

### Search strategy

A comprehensive search strategy was developed to encapsulate the current literature relevant to the topic **(Appendix A in** S2 File). The following subject headings and keywords were included: quality of healthcare, health equity, health stigma, health care accessibility, and sickle cell disease. Upon further development, a medical librarian trained in scoping review methods adapted the strategy to run across each of the seven database platforms. The initial search was conducted on 24 March 2023. To ensure recent publications were included, an update was conducted on 4 March 2024. Citation tracking was conducted from the included references to ensure a comprehensive search and within systematic reviews that were related to our topic but did not address our specific aim.

### Selection process

Data were exported from EndNote to Covidence, a web-based systematic review management program. Title and abstract screenings were conducted to determine if articles met the inclusion criteria. Following the preliminary screening, full-text review was conducted to confirm if all criteria were met and to exclude articles that did not meet the study criteria. All articles were subjected to thorough review and assessment by a minimum of two authors to reduce bias. Disagreements and/or conflicts were resolved by a third independent reviewer and final articles were ultimately chosen by consensus.

### Data collection

A pair of authors conducted a final assessment of all articles meeting the inclusion criteria. Data extraction was conducted using a Google form designed with inclusion and exclusion variables (see data items section for details on variables extracted).

### Data items

Study eligibility criteria determined extracted data. Additional data collected include study design, location (urban, rural, suburban), type of healthcare setting (school, hospital, clinic, community center, religious places of worships), inclusion criteria utilized, duration of the study, overall aim, total number of participants, gender, race, and age range of participants, control condition for the healthcare intervention (if applicable), experimental condition for a healthcare intervention (if applicable), duration of healthcare intervention (if applicable), reported barriers (if applicable, e.g.,. lack of insurance, racism, socio-economic), and any form of healthcare utilization (if applicable, e.g., any amount of emergency room visits, hospital visits, physician visits).

### Risk of bias assessment

The risk of bias (RoB) assessment was conducted on all included studies, using a modified Google Form. Depending on the study design, different RoB assessment tools were utilized to assess the studies. Randomized controlled trials (RCTs) were evaluated using the Cochrane RoB assessment [13]; Non-RCTs were evaluated based on Joanna Briggs Institute (JBI) [14]; Non-RCTs Cohort studies (Prospective, Retrospective, or Longitudinal or Observational) were evaluated based on the Newcastle–Ottawa Scale (NOS) [15]; Cross-sectional studies were evaluated based on JBI Critical Appraisal Checklist [16]; Case-Control studies were evaluated based on Critical Appraisal Skills Programme (CASP) checklist [17]; Qualitative studies were evaluated based on CASP as well [18]; and Mixed Method Studies were evaluated based on the Mixed Methods Appraisal Tool (MMAT) tool [19].

RoB was accessed in three categories following the specific assessment tool for each study design and their metrics of determination: Low RoB, High RoB, and Unclear/not applicable RoB. Low RoB indicated that the item on the assessment tool was described and well accounted for in the study, using the tool’s specifications for determination. High RoB indicated the item of bias was not sufficiently described and addressed in the study. Unclear/not applicable RoB indicated that there was no information provided in the study to determine if the item of bias was addressed.

Studies were then given an overall assessment on a scale ranging from 1 (poor-quality study and/or study indicates a high RoB) to 5 (high-quality study and/or study indicates a low RoB). Two independent reviewers assessed each study.

### Synthesis methods

Results were synthesized using (1) a descriptive table of the included studies, (2) a narrative table of study outcomes related to interventions utilized, barriers/facilitators, and healthcare utilization, (3) descriptive analyses such as frequency, (4) qualitative content analysis, and (5) narrative description of the syndemic coupling between healthcare barriers, facilitators, and utilization amongst individuals with SCD.

## Results

**Fig 1:**
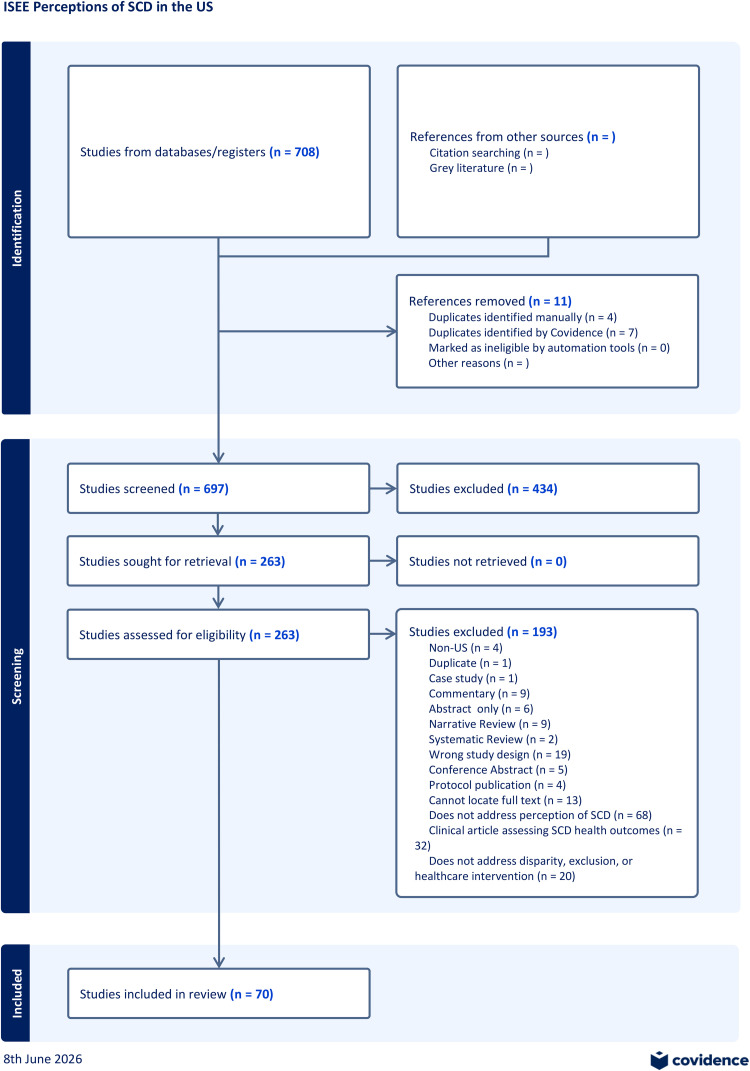
PRISMA study flowchart. This figure illustrates the systematic screening process used in the scoping review, applying predefined inclusion and exclusion criteria to identify eligible studies. Screening was conducted in two stages. In the first stage, titles and abstracts of 708 studies were screened, resulting in the exclusion of 434 records. In the second stage, full texts of 263 studies were retrieved and evaluated for eligibility. Of these, 70 studies were ultimately included in the final scoping review.

An initial search conducted on March 24, 2023, retrieved 668 articles and after removal of duplicates, 662 remained. The updated search on March 4, 2024, increased the number of articles to 708, with 697 yielding after removal of duplicates. Title and abstract screening further excluded 434 articles. 263 articles were reviewed, of which 193 were excluded and 70 were included. The screening, elimination process, and reasons for excluding articles are outlined in the PRISMA chart (Fig 1).

The studies included in this analysis consisted of a diverse sample size, ranging from 8 to 5,741,881 participants. All studies that disclosed demographic variables comprised of black participants (*n* = 70). Minors and young adults made up a large percentage of participation (*n* = 32). Studies were primarily conducted in urban locations (*n* = 29), a mix of urban and rural (*n =* 7), and a mix of urban, rural and suburban (*n* = 6). A large majority did not report where their study occurred in the U.S. (*n =* 23). Studies were reported to be conducted in the hospital (*n* = 30), the clinic (*n* = 16), both hospital and clinic (*n* = 7), and no report on settings (*n* = 8). Most of the studies were either from a prospective or retrospective cohort (*n* = 18), cross-sectional (*n* = 15) or mixed methods (*n* = 9). A description of all included studies is presented in **Table 1 and Table 2**.

**Table 1:** Description of Included Studies.

*Author, year*	*Study design*	*Study location*	*Study setting*	*Total sample size*	*Race/Ethnicity*	*Age range*
Alberts 2020 [20]	Mixed Methods	Urban	Clinic	149	Black; Hispanic; Other	Minor, Young Adult, Adult
Barriteau 2023 [21]	Cohort Studies (Prospective, Retrospective)	Suburban	Other: Illinois Health and Hospital Association, Comparative Health Care and Hospital Data Reporting Services (COMPdata)	79,779	Not Reported	Minor. Young Adult, Adult
Basu 2024 [22]	Other: Comparative Modeling Analysis	Not Reported	Not Reported; Other: Simulation models based on primary claims data	4762	White; Black; Hispanic; Not Reported; Other	Minor. Young Adult, Adult
Baumann 2023 [23]	Other: Qualitative	Urban; Suburban	School; Hospital	17	Not Reported	Young Adult, Adult. Middle Aged
Bediako 2011 [24]	Cohort Studies (Prospective, Retrospective)	Urban	Hospital; Clinic	95	Not Reported	Young Adult, Adult. Middle Aged, Aged
Bediako 2016 [25]	Cohort Studies (Prospective, Retrospective)	Urban	Clinic	262	Black	Young Adult, Adult, Middle Aged,
Benjamin 2000 [26]	Other: Comparative Study	Urban	Hospital	144	Black; Hispanic; Other	Other: Median age 30
Bergman 2013 [27]	Not Reported	Not Reported	Not Reported	N/A	Not Reported	Not Reported
Blakey 2023 [28]	Mixed Methods	Not Reported	Clinic	37	White; Black; Hispanic; Mixed Race	Young Adult, Adult, Middle Aged,
Boulet 2010 [29]	Not Reported	Not Reported	Not Reported	19,527	Black	Minor
Caldwell 2019 [30]	Cross-Sectional	Urban; Other: large, southern, metropolitan city	Clinic	134	Black	Minor, Young Adult
Calhoun 2022 [31]	Other: Qualitative	Urban	Other: conducted by an urban academic university	59	Black	Minor, Young Adult
Carroll 2009 [32]	Cohort Studies (Prospective, Retrospective)	Urban; Rural; Suburban	Hospital	122	Not Reported	Not Reported
Chestnut 1994 [33]	Other: Structured interview and survey (service perception test)	Not Reported	Hospital	55	White; Black	Minor, Adult
Crego 2020 [34]	Cohort Studies (Prospective, Retrospective)	Urban; Rural; Suburban	Hospital	2045	Not Reported	Minor, Young Adult, Adult, Middle Aged, Elderly
Crego 2021 [35]	Mixed Methods	Not Reported	Community Center	51	Black; Hispanic; Other: Do not know and missing	Adult
Crosby 2009 [5]	Other: Qualitative	Urban	Clinic	45	Black	Minor, Young Adult:
Desai 2020 [36]	Cohort Studies (Prospective, Retrospective)	Other: national	Hospital; Clinic	44,033	White; Black; Hispanic; Other	Minor, Young Adult, Adult, Middle Aged, Aged, Elderly
Edwards 2001 [37]	Cohort Studies (Prospective, Retrospective)	Not Reported	Not Reported	147	Black; Asian	Young Adult, Adult, Middle Aged,
Freiermuth 2014 [38]	Cohort Studies (Prospective, Retrospective)	Not Reported	Hospital	200	White; Black; Hispanic; Asian; Other	Not Reported
Goshua 2023 [39]	Other: Markov model	Not Reported	Hospital	N/A	Not Reported	Minor, Young Adult, Adult, Middle Aged, Elderly
Hankins 2012 [40]	Other: Pilot	Not Reported	Clinic	34	Not Reported	Minor, Young Adult:
Haque 2000 [41]	Other: Cross-sectional, secondary data	Urban; Rural	Other: Data for this study came from the clinical database of the Duke/University of North Carolina Comprehensive Sickle Cell Center	1298	Black	Not Reported
Hardy 2023 [42]	Cross-Sectional	Urban	Hospital	112	Not Reported	Minor
Haywood 2011 [43]	Cross-Sectional	Urban	Hospital; Clinic	94	Not Reported	Young Adult, Adult, Middle Aged, Aged
Haywood 2014 [44]	Cross-Sectional	Urban	Clinic	273	Not Reported	Minor, Young Adult, Adult, Middle Aged, Aged
Holdford 2021 [45]	Other: survey	Urban	Clinic	192	Black; Other	Not Reported
Jacob 2023 [46]	Cross-Sectional	Urban	Hospital	101	White; Black; Hispanic; Mixed Race	Not Reported
Jonassaint 2016 [47]	Cohort Studies (Prospective, Retrospective)	Urban	Hospital	258	Not Reported	Young Adult, Adult, Middle Aged, Aged, Elderly
Kam 2008 [48]	Cross-Sectional	Not Reported	Hospital	112	White; Black; Hispanic; Asian; Pacifier Islander; Mixed Race; Other: Missing	Minor
Kanter 2020 [49]	Other: Needs assessment survey	Urban; Rural	Clinic; Community Center	440	Black; Hispanic; Other	Minor, Young Adult, Adult; Middle Aged
Karras 2007 [50]	Not Reported	Urban	Hospital; Clinic	46	Not Reported	Minor, Young Adult, Adult
Kato-Lin 2014 [51]	Mixed Methods	Urban	Hospital	265	Not Reported	Minor, Young Adult, Adult, Middle Aged, Aged
Kirsch 2021 [52]	Not Reported	Urban	Hospital	58	Black; Hispanic; Other	Minor, Young Adult
Lanzkron 2008 [53]	Other: Questionnaire	Not Reported	Not Reported	48	White; Black; Hispanic; Asian	Not Reported
Lattimer 2010 [54]	Cohort Studies (Prospective, Retrospective)	Urban	Hospital	45	Not Reported	Young Adult, Adult, Middle Aged
Linton 2020 [55]	Cross-Sectional	Not Reported	Hospital	750	White; Black; Hispanic; Asian; Choose not to answer; Other: missing	Young Adult, Adult, Middle Aged,
Loo 2021 [56]	Other: Pilot	Urban; Rural; Suburban	Clinic	46	White; Black; Hispanic; Asian; Other	Young Adult, Adult, Middle Aged,
Mainous 2015 [57]	Other: survey	Not Reported	Not Reported	1042	White; Black; Hispanic; Asian; Other	Adult, Middle Aged:
Masese 2019 [58]	Mixed Methods	Urban; Rural; Suburban	Hospital	124	White; Black; Asian; Other: Missing	Adult, Middle Aged,
Mayo-Gamble 2020 [59]	Other: Community-based model	Urban; Rural	Clinic; Community Center; Temple/Church/Mosque	8	Black	Young Adult, Adult: Middle Aged
Molokie 2018 [60]	Cohort Studies (Prospective, Retrospective)	Urban	Hospital	148	White; Black; Hispanic; Mixed Race; Other: Non-Hispanic	Adult, Middle Aged
Mougianis 2020 [61]	Cross-Sectional	Urban	Hospital	75	Black; Hispanic; Mixed Race	Minor, Young Adult
Mupfudze 2021 [62]	Other: Qualitative	Not Reported	Not Reported	10	Not Reported	Not Reported
Nwogu-Onyemkpa 2022 [63]	Cross-Sectional	Not Reported	Hospital	987555	White; Black; Hispanic; Other	Young Adult, Adult, Middle Aged, Elderly
Panepinto 2012 [64]	Cohort Studies (Prospective, Retrospective)	Urban; Rural	Hospital	13533	Not Reported	Minor, Young Adult, Adult, Middle Aged, Elderly
Payne 2007 [65]	Cohort Studies (Prospective, Retrospective)	Not Reported	Hospital	49	Not Reported	Minor, Young Adult, Adult, Middle Aged
Pecker 2023 [66]	Other: Qualitative	Urban	Clinic	30	Not Reported	Young Adult, Adult, Middle Aged, Aged, Elderly
Perry Caldwell 2021 [67]	Cross-Sectional	Urban	Hospital; Clinic	75	Black	Minor, Young Adult
Peterson 2020 [68]	Cross-Sectional	Urban; Rural	Hospital	5,741,881	White; Black; Hispanic; Asian; Pacifier Islander; Other	Minor
Phillips 2022 [69]	Mixed Methods	Urban; Rural	Hospital	44	Black; Hispanic; Other: Non-Hispanic	Minor, Young Adult, Adult, Middle Aged
Power-Hays 2020 [70]	Cohort Studies (Prospective, Retrospective)	Urban	Clinic	132	Not Reported	Minor, Young Adult
Raphael 2013 [71]	Cross-Sectional	Urban	Hospital; Clinic	150	Not Reported	Minor
Ratanawongsa 2009 [72]	Cohort Studies (Prospective, Retrospective)	Urban	Hospital	131	White; Black; Hispanic; Asian; Pacifier Islander; Other	Young Adult, Adult, Middle Aged, Elderly
Schlenz 2022 [73]	Mixed Methods	Urban; Rural; Suburban	Clinic	51	White; Black; Hispanic; Asian; Other	Adult, Middle Aged, Aged
Shah 2019 [74]	Cohort Studies (Prospective, Retrospective)	Not Reported	Hospital; Clinic	3999	White; Black; Hispanic; Other	Minor, Young Adult, Adult, Middle Aged,
Shankar 2008 [75]	Cohort Studies (Prospective, Retrospective)	Urban; Rural; Suburban	Hospital	1,214	Not Reported	Minor, Young Adult
Shelley 1994 [76]	Cross-Sectional	Urban	Not Reported	11	Not Reported	Not Reported
Simmons 2019 [77]	RCT	Not Reported	Not Reported	34	Black	Young Adult, Adult, Middle Aged
Simpson 2017 [78]	Other: Pre- and post-implementation	Urban	Hospital	10	Not Reported	Not Reported
Smeltzer 2021 [79]	Cross-Sectional	Urban; Rural; Suburban; Other: 42 missing	Clinic	105	White; Black; Asian; Pacifier Islander; Choose not to answer; Other: 27 missing, 7 don’t know	Young Adult, Adult, Middle Aged, Other
Tanabe 2007 [80]	Cohort Studies (Prospective, Retrospective)	Not Reported	Hospital	159	White; Black; Hispanic	Young Adult, Adult, Middle Aged
Telfair 2003 [81]	Cross-Sectional	Urban; Rural	Clinic	662	Black	Minor, Young Adult, Adult
Udeze 2023 [82]	Not Reported	Not Reported	Hospital	20142	White; Black; Hispanic; Asian; Pacifier Islander; Other	Minor, Young Adult, Adult, Middle Aged, Elderly
Utrankar 2018 [83]	Mixed Methods	Urban	Hospital	86	Black; Other	Minor, Young Adult, Adult, Middle Aged,
Wakefield 2018 [84]	Mixed Methods	Urban	Hospital	20	Black	Minor, Young Adult,
Wesley 2016 [85]	Other: qualitative	Not Reported	Clinic	20	Black	Adult, Middle Aged
Wilkie 2010 [86]	RCT	Urban	Clinic	145	White; Black; Hispanic; Mixed Race	Young Adult, Adult
Williams-Gray 2015 [87]	Other: Qualitative	Urban	School	23	White; Black; Hispanic; Mixed Race	Young Adult, Adult, Middle Aged, Aged
Zhang 2021 [88]	Other: stepped-wedge trial design	Not Reported	Hospital	20628	Not Reported	Young Adult, Adult, Middle Aged

**For Age Range: Minors (Under 18); Young Adults (18–24); Adults (25–44); Middle Aged (45–64); Aged (65–79); Elderly (80+)**

**Table 2:** Description of Included Studies.

*Author, year*	*Study aim*	*Study duration*	*Main findings*
Alberts 2020 [20]	To design a mHealth intervention for individuals with SCD to improve adherence to hydroxyurea, using a user-centered design that was informed by specific barriers to hydroxyurea adherence and utilization in this population	Completion of all study phases took 1 year in total	The InCharge Health app is an mHealth intervention developed with substantial input from users and by mapping the HBM as the framework that guided the choice for its components
Barriteau 2023 [21]	To (1) describe ED visits and hospitalizations among IL residents with SCD over a 5-year period and (2) examine poverty level and other socioeconomic variables among individuals with SCD in IL who present for ED visits and hospitalizations	N/A	Our study demonstrates that individuals with SCD in IL have persistent high levels of ED visits and hospitalizations with little change over the last three decades
Basu 2024 [22]	To evaluate the cost-effectiveness of gene therapy for SCD and its value-based prices (VBPs)	N/A; Simulated models based on primary data	Gene therapy for SCD below a $2 million price tag is likely to be cost-effective when applying a societal perspective at an equity-informed threshold for cost-effectiveness analysis
Baumann 2023 [23]	To capture the implementation process of the ALIGN Study, (An individualized Pain Plan with Patient and Provider Access for Emergency Department care of Sickle Cell Disease	N/A	Findings contribute to learning how to implement E-IPPs for adult patients with SCD in ED. Findings highlight the importance of early engagement with different team members, a champion from the emergency department, study coordinators with different skills and enhancement of communication and trust among team members
Bediako 2011 [24]	To explore the relation between religious coping and hospital admissions in a sample of African American adults coping with sickle cell disease	2006 and 2007	These results indicate a need for further investigation of the roles that religion and spirituality play in adjustment to sickle cell disease and their influence on health care utilization patterns and health outcomes
Bediako 2016 [25]	To seek a more comprehensive understanding of SCD stigma by concurrently assessing external, internal, experienced, and anticipated domains	N/A	Three of the MoSCS factors (Social Exclusion, Internalized Stigma, and Expected Discrimination) were associated with patient-reported perceptions of disease severity and acute care visits for SCD pain, while two factors (Social Exclusion and Internalized Stigma) were associated with patient-reported hospital admissions for SCD pain
Benjamin 2000 [26]	To decrease the load of the emergency department and to study the value of a dedicated facility with knowledgeable staff applying principle-based individualized care	(1989-1993)	We conclude that a dedicated facility provides the kingpin for effective and rapid painful crisis management, reduces hospitalizations, and facilitates integration of the approach into other areas of care
Bergman 2013 [27]	To consider the relationship between the difficult patient- conundrum and SCD	N/A	By acknowledging the nuanced dimensions of sickle cell disease, we identify barriers to care particular to the disease, which further substantiate the need to redefine orthodox notions of the “difficult” patient
Blakey 2023 [28]	Sought caregivers’ and providers’ perspectives on processes underlying discrimination and potential solutions to mitigate the negative effects of perceived discrimination among children with SCD	March 2020 to March 2021	(1) healthcare system factors underlie discrimination, (2) families’ challenging interactions with providers lead to perceptions of discrimination, and (3) experiences of discrimination impact caregiver-provider interactions
Boulet 2010 [29]	To provide estimates of co-occurring conditions, health impact and utilization, and barriers to care for a national sample of children with SCD	NHIS from 1997 through 2005	The health burden for children with SCD and their families is profound and may be exacerbated by barriers to accessing comprehensive medical care
Caldwell 2019 [30]	To describe the relationship among health literacy levels, annual hospital encounters, annual clinic visits, annual ED visits, and annual hospitalizations in 134 black, non-Hispanic adolescents aged 10–19 years with SCD	October 2014 and September 2017	This study showed no significant relationships among health literacy, annual healthcare encounters, annual clinic visits, annual ED utilization and annual hospitalizations in adolescents with SCD
Calhoun 2022 [31]	To understand factors associated with optimal implementation of the AYA-SCD transition	1 year w/ focus group lasting b/w 45–80 mins. Interview lasted between 20 to 40 minutes	Our study highlights the importance of multi-level barriers and facilitators for AYA-SCD transition from pediatric to adult care
Carroll 2009 [32]	To describe the course of high inpatient utilization (averaging four or more admissions per year enrolled for at least one year) in members with a diagnosis of sickle cell disease and a history of hospitalizations for vaso-occlusive crisis	Not explicitly stated but 5 years was listed in one response	Members who were high utilizers had more diagnostic mentions of sickle cell complications than low utilizers. However, the pattern of high inpatient utilization was likely to moderate over successive years and return to the pattern after moderation was uncommon. Despite this, a small subpopulation engaged in exceptional levels of inpatient utilization over multiple years
Chestnut 1994 [33]	While strong ethnic loading of sickle cell is obvious, researchers have yet to explore how patients feel their ethnic and cultural characteristics influence the availability and quality of healthcare service they receive	N/A	Results from this study indicate race as being the most influential factor influencing health care delivery. Both medical staff and family respondents saw whites as getting better service than blacks
Crego 2020 [34]	To describe acute-care utilization in Medicaid-enrolled patients with SCD, patient factors associated with co management, and adherence to Hydroxyurea	1 year	Co Management was a factor in predicting HU adherence, but further studies are needed to identify the frequency and components of co management needed to increase adherence and reduce acute care utilization
Crego 2021 [35]	To use a multi-method approach to describe how patients with SCD in North Carolina perceive the care they receive in emergency departments	May to August 2017	Participant recommendations warrant further investigation to address persistent SCD quality of care concerns in the ED
Crosby 2009 [5]	To examine perceived barriers to clinic attendance and strategies to overcome these barriers for adolescents with sickle cell disease (SCD)	N/A	Adolescents with SCD and their families may benefit from on-going education about the importance of attending routine clinic visits
Desai 2020 [36]	Reporting age-specific rates of VOC events and healthcare utilization by assembling a large cohort of children and adults with SCD available using nationwide Medicaid claims between 2000 and 2013. Additionally, we also evaluated the influence of ongoing VOC burden on the risk of mortality in this cohort	1 year	In conclusion, we documented a substantial burden of SCD in US Medicaid enrollees, especially during early adulthood and noted that ongoing burden of VOC is associated with mortality in these patients
Edwards 2001 [37]	To examine (1) the relationships between self-efficacy and SCD adjustment at each time point, (2) the ability of baseline levels of self-efficacy to predict 1-year outcomes, and (3) the relationship between changes in self-efficacy over 1 year and changes in SCD adjustment over that same year	1 year	Self-efficacy beliefs among African American adults with sickle cell disease are inversely related to reported disease symptomatology, and these relationships persist across time
Freiermuth 2014 [38]	To validate a survey that measures attitudes towards patients with SCD among ED providers (nurses and physicians) and to compare differences in attitude scores between provider types	2 months	Provider attitudes influence patient-provider interactions and quality of care
Goshua 2023 [39]	To compare gene therapy versus standard of care (SOC) in patients with SCD by using conventional CEA and DCEA	12 months in duration w/ lifetime stimulation	Gene therapy is cost-ineffective per conventional CEA standards but can be an equitable therapeutic strategy for persons living with SCD in the United States per DCEA standards
Hankins 2012 [40]	To prepare adolescents for the transition process and establish a relationship with the adult care provider. The feasibility of the Transition Pilot Program was tested to investigate the ability of teenagers with SCD to participate in this program and the overall acceptance by adolescents, families, and health care providers. A secondary objective was to investigate the rate of fulfillment of first appointments with adult hematologists to provide some preliminary information about the program	Between June 2007 and December 2008	This transition pilot program was feasible, and most adolescent participants with SCD established an adult medical home
Haque 2000 [41]	To underscores the socioeconomic condition of patients with SCDs in North Carolina and the impact of that condition on their utilization of services	1991-1995	Utilization of services is directly related to socio economic conditions facing clients and clinic distance from clients. They further suggest that people in rural areas who ham high distress levels and are far from clinics haw limited access to health care
Hardy 2023 [42]	Whether neurocognitive and emotional factors predicted future pain-related healthcare utilization in children with SCD	3 years	Neurocognitive and emotional factors are associated with subsequent healthcare use in youth with SCD. Poor attentional control might limit implementation of strategies to distract from pain or could make disease self-management behaviors more challenging
Haywood 2011 [43]	To address the gap in current knowledge regarding SCD patient attitudes and beliefs about HU	September 2006 to June 2007	Barriers to a wider uptake of HU therapy among the SCD population exist at multiple levels, including at the level of the patient
Haywood 2014 [44]	To evaluate the association between perceived discrimination from healthcare providers and nonadherence to physician recommendations among persons with SCD, and to test the potentially mediating role of patient trust	2 years	SCD patient perceptions of discriminatory experiences from healthcare providers are associated with greater nonadherence to physician recommendations and may be a potential factor contributing to disparities in health and health quality among this patient population. Perceived discrimination appears to affect adherence behaviors through the pathway of patient trust
Holdford 2021 [45]	To quantify the indirect costs of sickle cell disease in the United States	June 2017 and December 2017	Sickle cell disease affected the work productivity, nonwork productivity, and the daily lives of adults seen with the disorder in an academic medical center
Jacob 2023 [46]	To understand the experiences of caregivers of pediatric patients in a geographically diverse area in the Midwest in accessing care, and their perspectives of telemedicine	N/A	The experiences of caregivers accessing healthcare for their child with SCD demonstrates significant caregiver burden and barriers to care regardless of nearness or accessibility of medical care
Jonassaint 2016 [47]	To determine what factors are related to emergency department (ED) visits in hopes of guiding treatments and early interventions	12-month period	Early interventions addressing disparities in academic performance, especially for those children most at risk, may lead to improved long-term health outcomes in this population
Kam 2008 [48]	To know how parents of Children with sickle cell disease perceive the quality of hospital care their children receive	June 2006 and August 2006	Parents of hospitalized children with sickle cell disease perceive their children’s care as being of lower quality than parents of children with cancer or children admitted to the general pediatric service
Kanter 2020 [49]	To assess the SCD-related medical care experience of adolescents and adults with SCD	July 2017 through March 2018	These results suggested that a negative perception of care may be a barrier for patients seeking care
Karras 2007 [50]	To learn directly from patients whether they perceived that their medical care was compromised eight months after Hurricane Katrina	May-23	Adult patients with SCD who relied almost exclusively on New Orleans main public hospital (Charity Hospital) for specialized sickle cell services reported significant frustration/dissatisfaction with their medical care eight months after the storm. In contrast, pediatric patients with SCD and their guardians, who rarely received care within the public hospital system, reported more satisfaction with their care
Kato-Lin 2014 [51]	To evaluate the impact of digitization of paper-based individualized pain plans on process efficiency and care quality by examining both objective patient data and subjective clinician insights	We define pre as January 1, 2008-April 30, 2010 and post as July 1, 2010-March 31, 2012	Highlights the important role of health information technology (HIT) on vaso-occlusive pain management for pediatric patients with sickle cell disease and the critical challenges in accommodating human factor considerations in implementing and evaluating HIT effects
Kirsch 2021 [52]	To examine the utilization, experience, and satisfaction of patients with Sickle Cell Disease (SCD) in the pediatric Emergency Department (ED) during the COVID-19 pandemic compared to the care they had received before the pandemic	February 2020-March 2021	Our results suggest that the COVID-19 pandemic significantly affected the decision of some patients as to whether and when to go to the ED
Lanzkron 2008 [53]	To evaluate provider’s awareness of the NHLBI recommendations regarding hydroxyurea prescribing, whether these recommendations have changed providers‚ practices and how these providers prescribed hydroxyurea	N/A	The results of this survey suggest that lack of awareness, agreement and belief in the benefits of hydroxyurea contributes to providers under prescribing hydroxyurea
Lattimer 2010 [54]	To measure the in-hospital experience of patients with SCD who received care during VOC and compare these experiences with those of a national sample of hospitalized patients	September 2006 to June 2007	Our study provides evidence that patients with SCD experience significant problems during hospitalization for VOC, especially in comparison with other hospitalized patients
Linton 2020 [55]	To identify institutional-, provider-, and patient-level barriers to care of sickle cell disease (SCD) in the emergency department (ED) to inform future interventions conducted by the multicenter Sickle Cell Disease Implementation Consortium	March 2017 and March 2018	The results underscore that many patients with SCD are dissatisfied with their ED care and highlight challenges to optimal care on the practice, provider, and patient levels
Loo 2021 [56]	To assess pediatric hematology clinic staff’s perspectives regarding barriers and facilitators in addressing unmet basic needs for children with sickle cell disease (SCD)	Between November and December 2019	These findings have important implications for how best to address adverse SDoH for this vulnerable pediatric population so that urban-based pediatric hematology clinics can more equitably support families
Mainous 2015 [57]	Our purpose was to examine family physician’s attitudes toward SCD management	Between November 2013 and January 2014	Family physicians are generally uncomfortable with managing patients and recognize the utility of CDS tools in managing patients
Masese 2019 [58]	To describe challenges and facilitators to caring for SCD from the perspective of ED providers in central North Carolina (NC)	June to September 2017	Illustrates the need for increased use of the NHLBI SCD recommendations, individualized pain protocols, and use of electronic medical records and other care-interventions, specifically geared towards improving provider knowledge and mitigating provider bias
Mayo-Gamble 2020 [59]	To develop and implement a SCD CHA training model (i.e., a statewide lay health advisor model) to effectively communicate to sickle cell community members on important issues on SCD and research	2 years	Study demonstrates the use of a CHA training model as an effective mechanism for recruiting and engaging the SCD community as partners in PCOR
Molokie 2018 [60]	To compare effects of usual care for adult SCD pain in ACU and ED on opioid doses and discharge pain ratings, hospital admission rates and lengths of stay	2 years	Applying guidelines for higher dosing of opioids for acute painful episodes in adults with SCD in ACU was associated with improved pain outcomes and decreased hospitalizations, compared to ED
Mougianis 2020 [61]	To explore associations among perceived racism, depressive symptoms, and HRQOL in adolescents with SCD	N/A	Findings suggest a model for how racism, depressive symptoms, HRQOL, and social support might interact in hospitalized adolescents with SCD
Mupfudze 2021 [62]	To evaluate alloHCT coverage and coverage of key benefits necessary for alloHCT, including routine patient care costs associated with clinical trials. This study also aimed to learn more about the experiences and perspectives of bone marrow transplant (BMT) financial coordinators working with their state Medicaid programs	June and November 2019	This study highlights the need for advocacy efforts at the state level aimed at passing legislative bills focused on increasing access to clinical trials and extending out-of-state coverage benefits to patients who need to enroll in out-of-state clinical trials
Nwogu-Onyemkpa 2022 [63]	To evaluate inpatient palliative care use during SCD-related hospitalizations overall and during terminal hospitalizations	January 2008 to 31 December 2017	Use of palliative care during SCD-related hospitalizations is increasing but remains low. Disparities associated with race and gender exist for use of palliative care services during SCD-related hospitalization
Panepinto 2012 [64]	To examine the concentration of hospital care for acute sickle cell disease-related visits. In addition, we studied the association between age and expected payer and the concentration of hospital care in patients with multiple acute sickle cell disease-related visits	2005-2006	Adults and patients with public insurance or no insurance are more likely to use multiple hospitals for acute care. By receiving acute care at multiple hospitals, patients with SCD experience dispersed and fragmented care potentially leading to decreased care quality
Payne 2007 [65]	1) to identify previously published estimates of the costs of ICT, 2) to update and improve these cost estimates by conducting a naturalistic cohort study to collect usual-care resource utilization data, and 3) to evaluate the real-world effectiveness of ICT, outside of the clinical trial environment, based on serum ferritin levels, adverse events, quality of life (QoL), and compliance to ICT	September 1 and November 1, 2005	Infused ICT may not provide adequate effectiveness in the real world. High ferritin levels seem to be associated with ICT noncompliance, likely in relation to the bothersome mode of administration and side effects. The total cost of ICT appears to well exceed that of drug alone
Pecker 2023 [66]	To elicit the perspectives of adults with SCD about their experience with telemedicine during the COVID-19 pandemic and to understand their preferences with respect to future telemedicine care	November 2020–March 2021	Telemedicine is an option that adults with SCD would like to see continue and that has the potential to expand access to care to more geographically distant regions
Perry Caldwell 2021 [67]	To evaluate health literacy in a cohort of 75 adolescents with sickle cell disease (SCD)	Between October and December 2014	Health literacy in adolescents with SCD is suboptimal
Peterson 2020 [68]	To analyze acute care utilization of sickle cell disease (SCD) and sickle cell trait (SCT) in children and identify trends in emergency department (ED) visits and inpatient admissions over a 10-year period	January 1, 2006 – September 30, 2015	Among patients less than 18 years of age with HbSS, inpatient admissions through the emergency department accounted for the largest medical expenditure of the SCD subtypes
Phillips 2022 [69]	To identify barriers to care from the perspective of individuals with SCD in a multi-state sample	March to June 2018	Participants reported several multilevel barriers to SCD care
Power-Hays 2020 [70]	To determine the feasibility of universal screening for SDoH in a busy subspeciality clinic using pre-existing resources, to identify the needs of our patients, and to facilitate referrals between our patients and community organizations	August 2017 – November, 2018	There is a high burden of SDoH in families of children with SCD. Universal screening in a pediatric hematology clinic with the subsequent connection of patients with SCD to community resources is feasible using existing clinic resources
Raphael 2013 [71]	To determine whether having a patient-centered medical home (PCMH) is associated with a reduction in emergency care (ED) utilization or hospitalizations among children with SCD	October 15, 2010, to May 4, 2011	Children with SCD reported to experience comprehensive care had lower rates of ED encounters and hospitalizations after controlling for demographics and health status
Ratanawongsa 2009 [72]	To investigate the validity and reliability of a scale to measure provider attitudes toward patients with acute VOC	September 2006 to June 2007	Our findings provide preliminary evidence for the reliability and construct validity of the PASS score in measuring provider attitudes toward patients with VOC
Schlenz 2022 [73]	To provide a comprehensive, multilevel examination of barriers and facilitators to transfusion therapy in children with SCA from health care provider and caregiver perspective	September 2018- March 2019	The comprehensive understanding of multilevel barriers and facilitators to transfusion therapy, including the role of nursing, in children with SCA can inform strategies to improve CRCT for patients/families and providers and can also be applied by organizations seeking to implement transfusion services for SCA
Shah 2019 [74]	To evaluate SCD treatment patterns and economic burden among patients prescribed hydroxyurea (HU) in the US, through claims data	January 1, 2009 – December 31, 201	The study showed the patients had significant unmet needs manifest as poor medication adherence, high treatment discontinuation rates, and high economic burden
Shankar 2008 [75]	To evaluate the impact of proximity to comprehensive care center on the healthcare utilization and mortality among children less than 20 years of age with SCD in the state of Tennessee, who were enrolled in TennCare, Tennessee Medicaid managed care program	January 1, 1995, to December 31, 2002	No clear pattern of improved utilization of medical care services were identified in relation to proximity of residence to a CSCC
Shelley 1994 [76]	To examine the activities that SCD self-help groups are undertaking to make changes in the delivery of health services	Nov-92	This study shows that in educating themselves and others about the experience of SCD, the members of the groups have taken a proactive role in their own health care which give them a sense of empowerment that they would not otherwise have
Simmons 2019 [77]	To examine the feasibility and acceptability of a telephonic MBI for adults with SCD and chronic pain	9 months	A MBI is feasible and acceptable for persons with SCD experiencing chronic pain
Simpson 2017 [78]	To address the healthcare needs of the vulnerable patient population, we piloted a multidisciplinary intervention seeking to create and use individualized patient care plans that alter utilization through coordinated care	Pre period was 06/01/13 to 12/31/13 (seven months) and post period was 01/01/14 to 02/28/15 (14 months).	A targeted approach is both feasible and potentially effective
Smeltzer 2021 [79]	To identify barriers to treating individuals with SCD.	N/A	A substantial number of providers did not know about NHLBI’s SCD care guidelines. Barriers to providing care for patients with SCD were influenced by providers’ specialty, training and practice setting
Tanabe 2007 [80]	To characterize the initial management of patients with sickle cell disease and an acute pain episode, to compare these practices with the American Pain Society Guideline for the Management of Acute and Chronic Pain in Sickle-Cell Disease in the emergency department, and to identify factors associated with a delay in receiving an initial analgesic	1 year	Patients with an acute painful episode related to sickle cell disease experienced significant delays to administration of an initial analgesic
Telfair 2003 [89]	To examine relationships between socioeconomic factors and the geographic distribution of 662 cases of sickle cell disease in Alabama in 1999–2001	August 1999 through March 2002	Conclusions based on statistical evidence that geographic location and socioeconomic factors relate to significantly different health care service experience bear important implications for medical and health care support systems, especially on the community level
Udeze, 2023 [82]	To describe the clinical complications, treatment use, healthcare resource utilization (HCRU), and costs among patients with sickle cell disease (SCD) with recurrent vaso-occlusive crises (VOCs) in the US	March 1, 2010, to March 1, 2019	Patients with SCD with recurrent VOCs experience substantial clinical and economic burden driven by inpatient costs and frequent VOCs
Utrankar 2018 [83]	To explore knowledge about guidelines; desire for guidelines; and how technology could support guideline awareness and adherence, examining current technology uses, and user preferences to inform design of a patient-centered guidelines application in a chronic disease	September 2016 to July 2017	Findings can inform the design of clinical practice guideline applications, suggesting a promising role for technology to engage patients, facilitate care decisions and actions, and improve outcomes
Wakefield 2018 [84]	To characterize racial bias events and reactions to these events of adolescents and young adults with SCD using mixed quantitative and qualitative methods	Between May 2012 and June 2014	Provides a description of racial bias experiences within community and medical settings and highlights the need for further evaluation of the impact of racial bias among youth with SCD
Wesley 2016 [85]	To explore the perception of stigma as reported by caregivers of adolescents with SCD	N/A	Stigma may affect individuals with SCD across multiple settings
Wilkie 2010 [86]	To describe sensory pain (location, intensity, quality and pattern), patient-related pain barriers, and the analgesics used by adult patients with SCD	N/A	Novel findings about the sensory pain characteristics and patient- related barriers reported by adult patients with SCD. Their barriers are greater than patients living with cancer
Williams-Gray 2015 [87]	To learn if appropriate health care for individuals with sickle cell disease, based on years of research, has been implemented	July 17, August 16, and October 15, 2012	After years of health care publications advocating procedures to be followed in the care of patients with SCD, there still appears to be a significant lack of connection between these recommendations of best practices and what is occurring in reality, at least in the New York City area
Zhang 2021 [88]	To examine the association between implementation of PDMP mandates and changes in opioids dispensed to these patients following ED encounters	N/A	Comprehensive PDMP mandates were associated with substantial reductions in opioids dispensed to patients with SCD or cancer with bone metastasis following ED encounters

### Healthcare barriers

Healthcare utilization barriers, as defined in this scoping review, are factors that prevent an individual, population and/or community from acquiring proper access to health services and subsequently achieving optimal quality of life [90]. Sixty-two out of the 70 studies included descriptions of healthcare barriers. Based on the data collected, there are significant barriers that impede PLWSCD from accessing health services (S1 Table). The top four barriers reported were social factors (*n* = 25), interpersonal issues (*n* = 23), economic factors (*n* = 15), and institutional level factors (*n* = 11).

Social determinants constituted the largest category of barriers, with stigma most frequently reported (*n* = 10), followed by racism (*n* = 8), bias (*n* = 5), discrimination (*n* = 4), and stereotyping (*n* = 2). Interpersonal barriers were primarily characterized by negative patient–provider relationships (*n* = 20) and poor patient perceptions of care (*n* = 3), reflecting the impact of implicit bias and stigmatizing attitudes within healthcare encounters.

**Fig 2:**
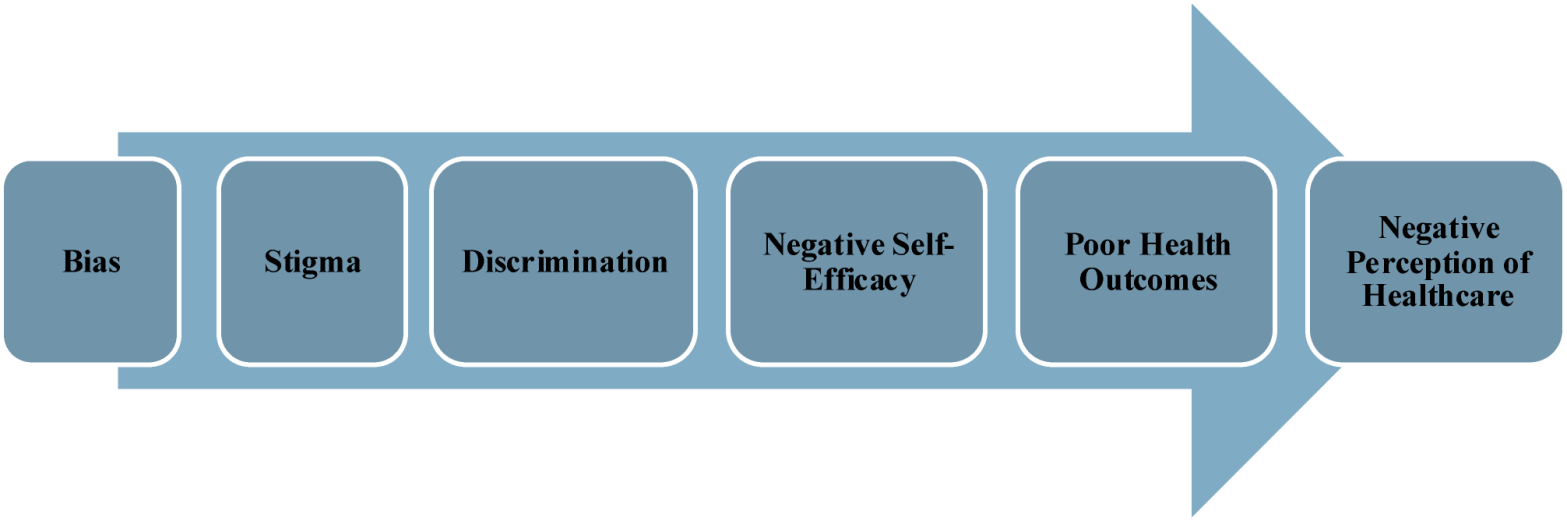
Interactions between racial dynamics, systemic oppression, and adverse health outcomes. The figure illustrates how race-related disparities and systemic oppression jointly contribute to adverse health outcomes and perceptions of healthcare**.** The blue arrow indicates a linear progression through which racial factors and marginalization influence these outcomes.

**Fig 2** displays the complex interplay between race, expressions of oppression and negative health outcomes – demonstrating how race-related differences collectively lead to a negative perception of healthcare.

**Fig 3:**
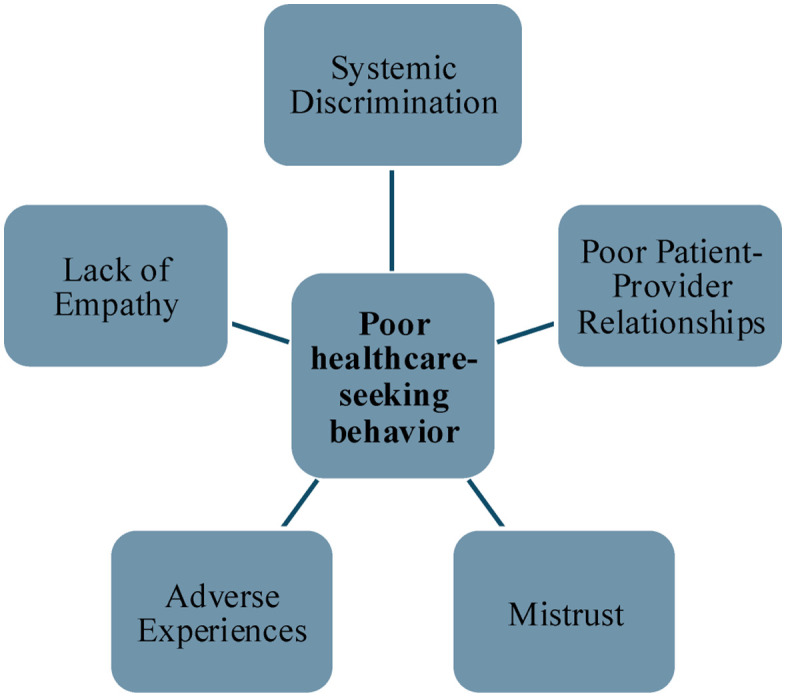
Institutional-level barriers to healthcare utilization. The figure illustrates healthcare-system related factors that contribute to poor healthcare seeking behavior among individuals with SCD.

Economic and institutional barriers further compounded these inequities. Economic challenges included high upfront healthcare costs (*n* = 6) and lack of transportation (*n* = 3) which disproportionately affect individuals of lower socioeconomic status. Institutional-level barriers, though less frequently reported, included administrative and operational inefficiencies (*n* = 2), such as overcrowded emergency departments, prolonged wait times, lack of SCD-specific care protocols, poor provider communication, and shared clinical resources with other specialties, all of which delay timely treatment during acute crises [5,8,91,55]. In outpatient settings, limited clinic availability, inadequate care coordination, and high staff turnover disrupt continuity of care, particularly in rural and underserved areas [10,14,91,63,87,33]. Characterization of institutional level barriers to healthcare utilization are displayed in **Fig 3**.

Additionally, reliance on administrative data and case-finding algorithms contributes to inaccuracies in patient identification and healthcare utilization estimates, undermining effective resource allocation and service planning [57]. The cumulative impact of these institutional inefficiencies intersects with socioeconomic disadvantage, including limited access to insurance, food insecurity, and inability to meet basic needs [35,43]. Given the substantial lifetime cost of SCD care — estimated at $1.6 million — and significant productivity losses due to employment disruption and chronic pain [41,38,27], racial discrimination, low socioeconomic status, and systemic inefficiencies together form a self-reinforcing cycle of poor healthcare experiences and adverse health outcomes for PLWSCD (S2 Table).

### Healthcare facilitators

Healthcare facilitators, as defined in this scoping review, are factors that favor or help an individual’s, population’s and/or community’s accessibility to healthcare services [92]. Twenty-five out of the 70 studies mentioned facilitators. Based on the data collected, the top four facilitators mentioned were technology (*n* = 9), education (*n* = 7), autonomy (*n* = 6), and a positive patient-provider relationship (*n* = 6). Characterization of the studies that discussed healthcare facilitators are presented in (S2 Table).

These facilitators play a crucial role in mitigating the barriers discussed in the previous section. Education, self-advocacy, and community support emerged as key elements; health literacy and educational interventions empower PLWSCD to manage their condition, communicate effectively with providers, and actively participate in care decisions. Peer and community support mitigate isolation, foster emotional resilience, and encourage leadership as well as civic engagement [10,16,93,30,63,27,34,59,40,52].

Technology complements these efforts by providing tools such as reminders, tracking systems, and clinical decision support, which enhance adherence, care coordination, self-efficacy, and personalized care for both patients and providers [93,32,30,63,59,80,94]. Together, education, technology, autonomy, and supportive patient–provider relationships were demonstrated to strengthen self-management, improve care quality, and facilitate more equitable SCD care.

**Fig 4:**
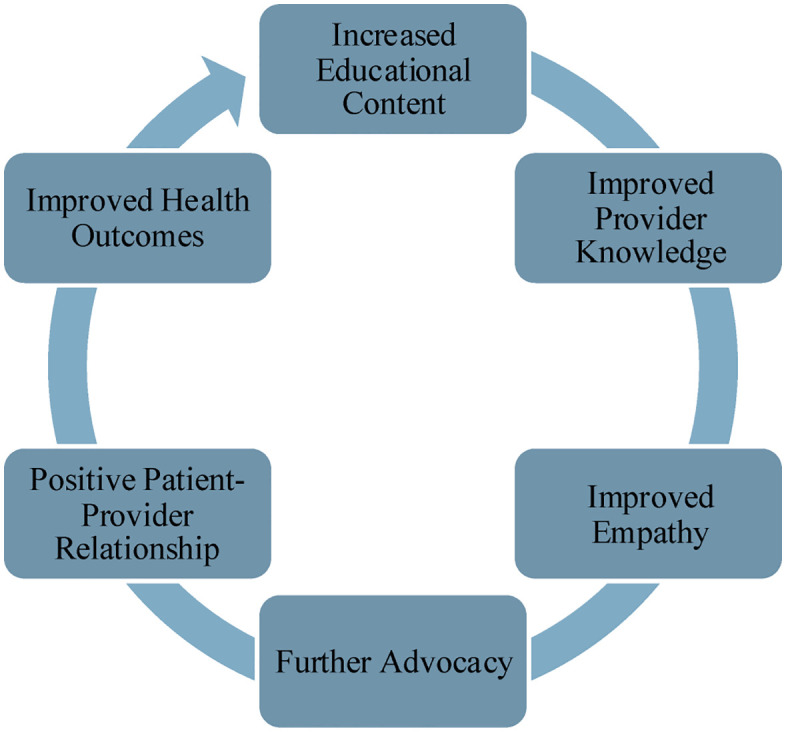
Provider-level facilitators supporting optimal management of SCD. The blue arrow represents a linear progression demostrating how cumulative steps by healthcare providers contribute to reducing barriers to optimal SCD management.

**Fig 5:**
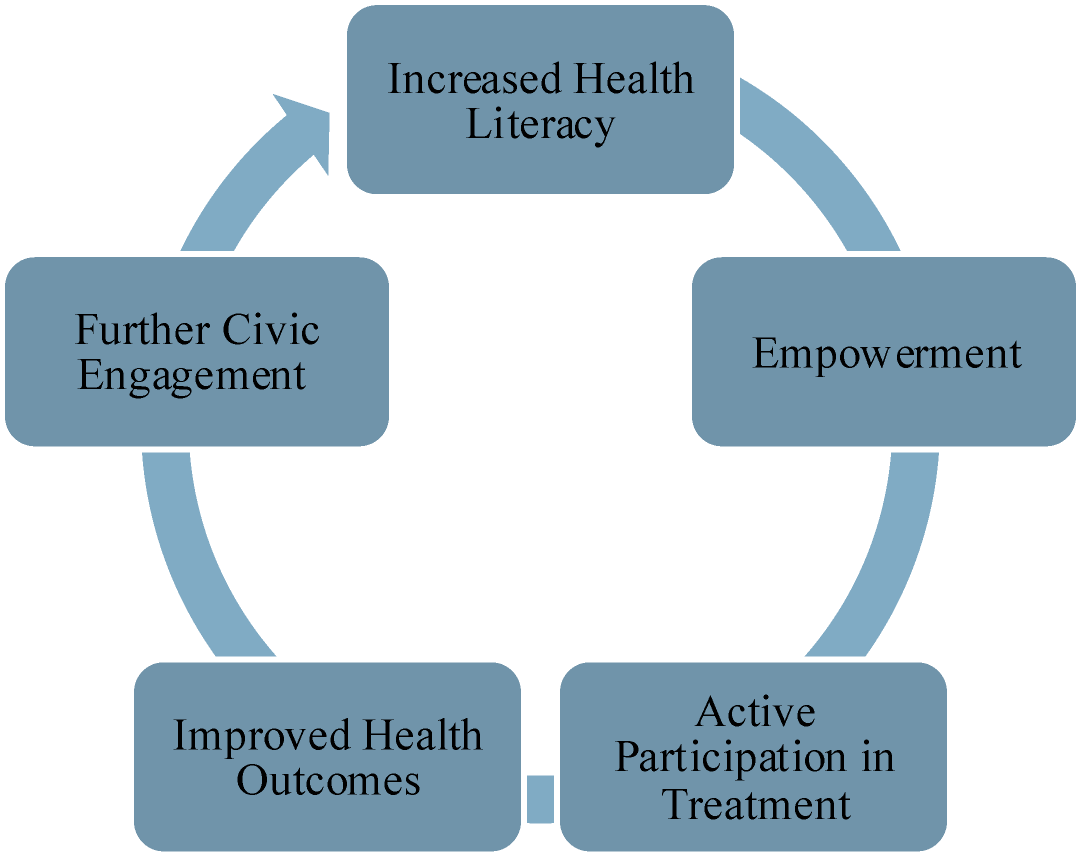
Individual-level facilitators supporting optimal management of SCD. The blue arrow represents a linear progression demostrating how sequential patient-level actions and resources contribute to reducing barriers to optimal SCD management.

**Figs 4 and 5** illustrate the characterization of these facilitators that play a crucial role in mitigating the barriers identified in the previous section. A linear progression (indicated by the blue arrow) demonstrates how cumulative steps can decrease impediments in relation to optimal management of SCD.

### Healthcare utilization

Healthcare utilization, as defined in this scoping review, is the usage of health-care services to maintain well-being or to obtain further information regarding individual, population and/or community health status [95]. Forty-seven out of the 70 studies included descriptions of healthcare utilization. The most common forms of healthcare utilization mentioned were hospital admissions (*n* = 19) and ED-visits (*n* = 18), followed by clinic visits (*n* = 7), therapy (*n* = 7), and outpatient (*n* = 6) with gene therapy (*n* = 2) being the most frequent form of therapy. Characterization of the studies that discussed healthcare utilization is in (S3 Table).

These patterns highlight that PLWSCD rely heavily on both emergent and non-emergent services due to chronic pain, stress, and complications from SCD [6,7,9,14,96,28,33,76,85,87,61,44,49,79,48,50,73,84,97]. In particular, frequent usage of the ED reflects limited access to preventive care, flexible access to clinics, and scheduling challenges or barriers to timely appointments [5,7,15,95,96,35,76,44,49,55,79,83,21,45,82,84,89,97]. Fragmented care – driven by multiple hospital visits, insurance limitations or lack of, and provider knowledge gaps – reduces care quality and contributes to high readmission rates of roughly 30% [54,48,22]. Inadequate pain management, poor coordination, and prior negative experiences also increase reliance on emergent care [6, 23, 43, 48}. The Covid-19 pandemic further disrupted routine care but accelerated the usage of telemedicine, which improved access and efficiency [61,39].

### Healthcare interventions to increase access to care for patients with SCD

Healthcare interventions as defined in this scoping review are non-pharmacological or pharmacological means intended to act on the health of an individual, population and or community to encompass health promotion, prevention, and protection [93]. Eight out of the 70 studies included descriptions of healthcare interventions. The most common forms of interventions were individualized pain plans (IPPs) (*n* = 4) and strategies designed for quality improvement (QI initiatives) (*n* = 3). The remaining one was a non-pharmacological intervention designed to reduce pain (*n* = 1). Characterization of the studies that discussed healthcare interventions is presented in (S4 Table).

### Risk of bias assessment findings

Final assessment of the RoB showed that 21.43% (*n* = 15) received an overall assessment of 5; 52.86% (*n* = 37) received an overall assessment of 4; 10.00% (*n* = 7) received an overall assessment of 3; 8.57% (*n* = 6) received an overall assessment of 2; and 7.14% (*n* = 5) received an overall assessment of 1.

Studies that did not report a design were automatically given a poor assessment since the absence of thorough description of the study’s methodology, data collection, and sufficient analysis limits the study’s objectivity. Studies that did report a design but did not have a designated framework were assessed based on the overall quality.

The average assessment was 3.73, indicating that there is a slight RoB within the results reported in the studies. One major source of bias that may have affected outcome reporting is that most of the studies within the cross-sectional and cohort design utilized self-reporting. Within the cohort design, complete follow-up or follow-up rate was not mentioned. The studies within the RCT design (*n* = 2) did not have allocation concealment, blinding of personnel and/or blinding of participants. The RoB assessment is characterized in (S5 Table).

## Discussion

The root of healthcare avoidance is very complex and deeply rooted in a web of social, psychological, and structural determinants. Albert Bandura’s Social Cognitive Theory (SCT) [39] provides an illuminating lens through which we can analyze the intricate interplay between individuals and their environment [91]. The SCT posits that behavior – such as the decision to seek or avoid care – is shaped not only by personal factors, but also by the environmental contexts in which behaviors are learned and reinforced (e.g., experiences within schools, clinics, and hospitals) [91]. The theory also acknowledges the lasting influence of previous experiences – whether fulfilling or traumatic – in shaping current behaviors and attitudes [91]. Our scoping review highlights this perspective by identifying how factors such as negative healthcare experiences, clinical bias, and mistrust shape the expectations of individuals and subsequently influence their decision to either engage or disengage from the healthcare system.

As a complement to the SCT, the Health Belief Model (HBM) deepens the understanding of health-related decision making by focusing on individual perceptions and beliefs [96]. Within the HBM, healthcare-withdrawing behavior is influenced by perceptions of the quality and reliability of care (e.g., encountering poorly influenced clinicians or dismissive medical teams), internalized beliefs such as self-stigma or fear of mistreatment, and an individual’s sense of self-efficacy [96].

Evaluations regarding self-efficacy often extend beyond medical consequences (e.g., disease progression or further complications) to include anticipated social impacts (e.g., disruptions to family life and intimate relationships) that dictate their confidence in navigating the healthcare system. [96]. Therefore, for individuals who opt against seeking treatment or adopting disease prevention strategies, the decision to delay or completely avoid care is rooted in a rational evaluation of perceived barriers versus anticipated benefits. Our scoping review echoes this statement by exhibiting how individuals calculate the emotional and logistical costs of seeking care with consideration of socio-economic circumstances, emotional turmoil, mistrust, and stigma associated with SCD against the perceived benefits of care.

Alternatively, external cues can offer alleviation of internalized stigma and increase motivation for healthcare-seeking behavior. The emphasis on self-efficacy and a cue to action [96] – triggers that prompt individuals to take health-related options – is reflected in our scoping review’s identification of facilitators such as medical staff advocacy, community-based initiatives and targeted education.

By anchoring our findings to the theoretical frameworks of the SCT and the HBM model, it remains evident that addressing the barriers to care for individuals with SCD require a multi-level strategy that enhances self-efficacy, reshapes environmental influences, and recalibrates individual beliefs about healthcare. Our scoping review identifies a constellation of barriers that encompass social, interpersonal, economic, and institutional level factors — all of which that compound to significantly impede healthcare utilization among individuals with SCD within the U.S. In contrast, facilitators at both an interpersonal (e.g., strong system of support) and intrapersonal (e.g., self-advocacy) levels served as antitheses to the barriers found. While our findings support the existing literature on the ongoing systemic gaps in healthcare for people with SCD [6], this scoping review contributes novel insights that (1) address the top aforementioned barriers and facilitators to healthcare utilization and (2) offering a comprehensive mapping of the literature including interventions designed to address such obstacles.

Correspondingly, the interventions reviewed specifically target the structural and behavioral barriers identified by amplifying the role of the facilitators outlined, thus offering actionable pathways in operationalizing the principles of the SCT and the HBM model to reduce health disparities and improve care engagement within the SCD population.

By understanding the perspectives of those that are most impacted, strength-based approaches can improve medical guidelines while fostering a sense of autonomy. EBIs, such as pain-based care and psychosocial support, have demonstrated potential in reducing healthcare avoidance and improving health outcomes. Nevertheless, the predominant reliance on ED visits and hospital admissions over preventative care highlights the urgent need for improved access to non-emergent healthcare services. Furthermore, the strength of evidence varies across the reviewed studies, with notable limitations including heterogeneity in study designs, populations, and outcome measures. Additionally, there is a need for longitudinal studies to examine the long-term effects of interventions on healthcare utilization and health outcomes in PLWSCD.

### Strengths and limitations

This study used the PRISMA framework to review literature documenting the perception of SCD, including healthcare barriers, healthcare utilization and interventions used for individuals suffering from such a chronic disease. Additionally, no study designs were restricted to ensure a full sweep of published data. Relevant information from the observational studies was captured utilizing a double screening and double data entry methodology. Studies that were included were restricted to English-only or available translations of non-English articles. It is possible that the review omitted studies not published in English. Additionally, the utilization of self-reporting within the cross-sectional and cohort design may have affected the validity of the outcomes due to under-reporting, over-reporting, or memory lapses. Failure to mention complete follow-up or follow-up rates in cohort studies may have lent itself to bias and under-valuation of confounding effects. Similarly, the lack of randomization and the overall anonymity of the participants to the research staff when conducting the final physiological measurements for the RCTs may have contributed to various sources of bias. Nonetheless, the use of these metrics may have been impossible given the scope of the research question and the nature of the studies themselves

Further limitations may be due to excluding non-U.S. based studies including studies from low-and middle-income countries. Thus, the critical need for comprehensive strategies to address the barriers that PLWSCD face may be highly underestimated in this scoping review since it does not account for non-U.S. studies. There is paucity of research and interventions targeting the underserved SCD population that address healthcare barriers, compromising efforts to improve healthcare access and overall health outcomes for this population.

### Conclusion

This scoping review provides an overview of the barriers and facilitators influencing healthcare utilization among individuals with SCD in the U.S. Our review also emphasizes the critical need to address these systemic barriers through the implementation of targeted EBI. The primary barriers identified include socio-economic challenges, stigma, and institutional obstacles at the clinical level. These significant barriers to healthcare access and utilization adversely affect healthcare outcomes for PLWSCD. The facilitators found were nearly unanimously contrasted with the barriers mentioned in this scoping review at the individual, provider and organizational level.

Future research should focus on the following: (1) enhancing patient experience and self-autonomy, (2) endorsing support systems and multidisciplinary approaches to streamline SCD care, (3) improving health literacy through tailored SCD education, and (4) applying a dual perspective approach with contextual lens to champion health equity (i.e., engage both healthcare professionals and patients / caregivers in developing contextually relevant interventions).

Despite the availability of EBIs for SCD management in the U.S., not all interventions may be accessible to PLWSCD of low SES. Not only do these individuals have poorer clinical outcomes, but they also face additional barriers that hinder their ability to maximize outcomes following the implementation of the interventions. The interventions recommended should be contextually relevant to ensure that the challenges faced in receiving or delivering the intervention are fully addressed. While it is crucial to consider the perspectives of those delivering the interventions, it is equally important to investigate the views of those on the receiving end [98]. Focusing on issues solely identified by the delivery team may not result in designing an intervention that is acceptable or beneficial to PLWSCD [98]. By including both the perspectives of the professionals and beneficiaries, advocates can (1) gain a deeper understanding of the specific needs of members from low SES communities and (2) design more successful and tailored interventions [98]. Fostering a healthcare environment that supports the unique needs of PLWSCD is vital for significantly improving clinical outcomes and quality of life. This dual perspective approach ensures that interventions are practical and resonate with the lived experiences of SCD sufferers, leading to more effective and sustainable healthcare solutions that uphold equity.

### Definitions

Perception: we used the below definitions to capture the perception of healthcare utilization from PLWSCD

Healthcare Barriers: factors that prevent an individual, population and/or community from acquiring proper access to health services and subsequently achieving an optimal quality of life

Healthcare Facilitators: factors that favor or help an individual’s, population’s and/or community’s accessibility to healthcare services.

Healthcare Utilization: the use of health-care services to maintain well-being or to obtain information regarding individual population and/or community health status.

Healthcare Intervention: Non-pharmacological or pharmacological means intended to act on the health of an individual, or community to encompass including health promotion, prevention, and protection.

## Supporting information

S1. File PRISMA checklist. (DOCX)

S2. File Appendix A: PubMed full search strategy. (DOCX)

S1 Table Barriers to SCD management reported by studies. (DOCX)

S2 Table Facilitators to SCD management reported by studies. (DOCX)

S3 Table Healthcare access/Utilization reported by studies. (DOCX)

S4 Table EBIs reported by studies. (DOCX)

S5 Table RoB assessment. (DOCX)

## Abbreviations

SCD: Sickle cell disease
PLWSCD: People living with SCD
U.S.: United States
RoB: Risk of Bias
SCA: Sickle Cell Anemia
ED: Emergency Department
ER: Emergency Room
EBI: Evidence-Based Intervention
IPP: Individualized Paper Plan
QI: Quality Improvement
SES: Socio-economic Status
SDoH: Social Determinants of Health
EHR: Electronic Health Records
HU: Hydroxyurea.
