# Supplementary material for: Barriers and facilitators to healthcare utilization amongst people living with sickle cell disease in the United States: A scoping review
Source: PLoS One. 2026 Jul 6;21(7):e0349441. doi: 10.1371/journal.pone.0349441 (PMC13336462; doi:10.1371/journal.pone.0349441)
Supplement: S1. File — (DOCX) [file pone.0349441.s001.docx]

**Preferred Reporting Items for Systematic reviews and Meta-Analyses extension for Scoping Reviews (PRISMA-ScR) Checklist**

| **SECTION** | **ITEM** | **PRISMA-ScR CHECKLIST ITEM** | **REPORTED ON PAGE #** |
| --- | --- | --- | --- |
| **TITLE** | | | |
| Title | 1a | Identify the report as a scoping review | 1 |
| **AUTHORS** | | | |
| Authors | 1b | List all authors and responsibilities (if possible)  ***Christina Ruan, Joyce Gyamfi, Nana Osei-Tutu, Shreya Meda, Lydia Samuels, Nousheen Inayat, Sukruthi Thunga, Elizabeth Noble, Dorice L. Viera, Deborah Adenikinju, Charmaine Royal, Angela Odoms-Young, Prince Michael Amegbor, Emmanuel Peprah*** | 1 |
| **ABSTRACT** | | | |
| Structured summary | 2 | Provide a **brief** structured summary that includes (as applicable): background, objectives, eligibility criteria, sources of evidence, charting methods, results, and conclusions that relate to the review questions and objectives.  Introduction  Methods  Results (after completion of analysis)  Discussion | 2-3 |
| **INTRODUCTION** | | | |
| Rationale | 3 | Describe the rationale for the review in the context of what is already known. Explain why the review questions/objectives lend themselves to a scoping review approach.  ***Given that there is a decrease in the number of individuals seeking care under a physician, there is common agreement that those with chronic conditions subsequently shy away from seeking care. Black children who suffer from SCD have been found to be in the hospital for >40 days, bringing us to the conclusion that their symptoms are poorly managed and that their avoidance towards the healthcare system stems from stigma, structural racism, etc. Therefore, this review is seeking to understand the interaction between the number of doctor visits and maternal education (above high school) by answering the following questions: 1)Why are these individuals less likely to seek medical care and 2?What are the structural factors?*** | 5-6 |
| Objectives | 4 | Provide an explicit statement of the questions and objectives being addressed with reference to their key elements (e.g., population or participants, concepts, and context) or other relevant key elements used to conceptualize the review questions and/or objectives.  ***This scoping review will look what barriers and/or facilitators impact decrease utilization of healthcare among those affected by SCD*** | 6 |
| **METHODS** | | | |
| Protocol and registration | 5 | Indicate whether a review protocol exists; state if and where it can be accessed (e.g., a Web address); and if available, provide registration information, including the registration number.  ***https://doi.org/10.17605/OSF.IO/KUAZW*** | 7 |
| Eligibility criteria | 6 | Specify characteristics of the sources of evidence used as eligibility criteria (e.g., years considered, language, and publication status), and provide a rationale.  ***Inclusion: SCD, perceptions as it relates to healthcare/frequency of ER/hospitals, U.S. studies***  ***Exclusion: Non-U.S. studies*** | 7 |
| Information sources* | 7 | Describe all information sources in the search (e.g., databases with dates of coverage and contact with authors to identify additional sources), as well as the date the most recent search was executed.  ***PubMed/Medline, Embase, Cochrane Library, Global Health, Web of Science (all databases), CINAHL, SCOPUS, grey literature, WHO/UN websites, eTable of Contents, and bibliography review*** | 8 |
| Search | 8 | Present the full electronic search strategy for at least 1 database, including any limits used, such that it could be repeated.  **(perception* [mesh] OR perception [tw] OR perspectives [tw] OR beliefs [tw]) OR (barriers OR facilitators OR disparities OR obstacles OR health equity OR access to medication OR resource limited OR burdens OR complications OR stigma OR discrimination OR racial bias OR psychosocial stressors OR non-compliance OR financial challenge) AND (health care accessibility OR health care utilization OR patient access OR quality of assistance OR patient advocacy OR quality of care OR health services) AND (sickle cell disease [tw] OR sickle cell anemia [tw] OR Hemoglobin S disease [tw]) AND (united states [mesh] OR united states [tw])** | 8, S2 File (Appendix A) |
| Selection of sources of evidence† | 9 | State the process for selecting sources of evidence (i.e., screening and eligibility) included in the scoping review.  ***Covidence, a web-based program for managing literature reviews will be used for dual screening, full text assessment, and data extraction. Excel will be used for data synthesis.*** | 8 |
| Data charting process‡ | 10 | Describe the methods of charting data from the included sources of evidence (e.g., calibrated forms or forms that have been tested by the team before their use, and whether data charting was done independently or in duplicate) and any processes for obtaining and confirming data from investigators.  ***Using Excel’s Pivot Tables, data will be charted and summarized.*** | 9 |
| Data items | 11 | List and define all variables for which data were sought and any assumptions and simplifications made.  ***Study design, location, setting, aim, inclusion criteria, and duration***  ***Participant numbers, gender, race, and age***  ***Control and Experimental Conditions for Healthcare Intervention (If applicable to study)***  ***Facilitators (If applicable to study)***  ***Barriers (If applicable to study)***  ***Type of Health Care Facility***  ***Main Findings of Study*** | 9 |
| Critical appraisal of individual sources of evidence§ | 12 | If done, provide a rationale for conducting a critical appraisal of included sources of evidence; describe the methods used and how this information was used in any data synthesis (if appropriate).  ***Because the PICO question addresses a clinical topic- Sickle Cell Disease- we used standard tools for evaluating risk of bias based on study design*** | 9-10 |
| Synthesis of results | 13 | Describe the methods of handling and summarizing the data that were charted.  ***Synthesis will be based on pivot tables and descriptive narrations of study outcomes*** | 10 |
| **RESULTS** | | | |
| Selection of sources of evidence | 14 | Give numbers of sources of evidence screened, assessed for eligibility, and included in the review, with reasons for exclusions at each stage, ideally using a flow diagram. | 10-11, S3 File (Appendix B) |
| Characteristics of sources of evidence | 15 | For each source of evidence, present characteristics for which data were charted and provide the citations.  ***Study characteristics will be listed under “results”*** | 11, S1 Table |
| Critical appraisal within sources of evidence | 16 | If done, present data on critical appraisal of included sources of evidence (see item 12).  ***RoB assessment***  ***RoB findings*** | 9-10, 13-14, 42-43 S6 Table |
| Results of individual sources of evidence | 17 | For each included source of evidence, present the relevant data that were charted that relate to the review questions and objectives.  ***Healthcare barriers***  ***Healthcare facilitators***  ***Healthcare utilization***  ***Healthcare interventions to increase access to care for patients with SCD*** | 11-13, S2, S3, S4, S5 Tables |
| Synthesis of results | 18 | Summarize and/or present the charting results as they relate to the review questions and objectives.  ***Descriptions will be provided under the respective source of evidence*** | 11-13, S2, S3, S4, S5 Tables |
| **DISCUSSION** | | | |
| Summary of evidence | 19 | Summarize the main results (including an overview of concepts, themes, and types of evidence available), link to the review questions and objectives, and consider the relevance to key groups.  ***Healthcare barriers are a challenge to health equity: the interplay of discrimination and socioeconomic barriers in SCD***  ***Unveiling the hidden barriers: administrative and operational challenges in SCD care access***  ***Healthcare utilization patterns in PLWSCD***  ***SES disparities inaugurated in SCD care***  ***Understanding patient perspective and provider biases in SCD care***  ***Facilitators on the provider level in SCD care***  ***Facilitators on the Individual and the role of community in SCD care***  ***Education and technology as facilitators in SCD care***  ***The role of health literacy and autonomy utilizing healthcare transition for PLWSCD as an case exemplar***  ***Bypassing the status quo: establishing alternative care strategies for PLWSCD***  ***Enhancing SCD care through IPPs*** | 14-42 |
| Limitations | 20 | Discuss the limitations of the scoping review process.  ***Limitations may be due to the exclusion of non U.S. studies*** | 45-46 |
| Conclusions | 21 | Provide a general interpretation of the results with respect to the review questions and objectives, as well as potential implications and/or next steps.  ***Future research should focus on the following: (1) enhancing patient experience and self-autonomy, (2) endorsing support systems and multidisciplinary approaches to streamline SCD care, (3) improving health literacy through tailored SCD education, and (4) applying a dual perspective approach with contextual lens to champion health equity*** | 43-35 |
| **FUNDING** | | | |
| Funding | 22 | Describe sources of funding for the included sources of evidence, as well as sources of funding for the scoping review. Describe the role of the funders of the scoping review.  ***Not applicable*** | N/A |

JBI = Joanna Briggs Institute; PRISMA-ScR = Preferred Reporting Items for Systematic reviews and Meta-Analyses extension for Scoping Reviews.

* Where *sources of evidence* (see second footnote) are compiled from, such as bibliographic databases, social media platforms, and Web sites.

† A more inclusive/heterogeneous term used to account for the different types of evidence or data sources (e.g., quantitative and/or qualitative research, expert opinion, and policy documents) that may be eligible in a scoping review as opposed to only studies. This is not to be confused with *information sources* (see first footnote).

‡ The frameworks by Arksey and O’Malley (6) and Levac and colleagues (7) and the JBI guidance (4, 5) refer to the process of data extraction in a scoping review as data charting*.*

§ The process of systematically examining research evidence to assess its validity, results, and relevance before using it to inform a decision. This term is used for items 12 and 19 instead of "risk of bias" (which is more applicable to systematic reviews of interventions) to include and acknowledge the various sources of evidence that may be used in a scoping review (e.g., quantitative and/or qualitative research, expert opinion, and policy document).

*From:* Tricco AC, Lillie E, Zarin W, O'Brien KK, Colquhoun H, Levac D, et al. PRISMA Extension for Scoping Reviews (PRISMAScR): Checklist and Explanation. Ann Intern Med. 2018;169:467–473. [doi: 10.7326/M18-0850](http://annals.org/aim/fullarticle/2700389/prisma-extension-scoping-reviews-prisma-scr-checklist-explanation).
