# Supplementary material for: Barriers and facilitators to healthcare utilization amongst people living with sickle cell disease in the United States: A scoping review
Source: PLoS One. 2026 Jul 6;21(7):e0349441. doi: 10.1371/journal.pone.0349441 (PMC13336462; doi:10.1371/journal.pone.0349441)
Supplement: S2. File — (DOCX) [file pone.0349441.s002.docx]

**S2 File. Appendix A: Search Strategy**

(perception OR perspectives OR beliefs) AND (barriers OR facilitators OR disparities OR obstacles OR health equity OR access to medication OR resource limited OR burdens OR complications OR stigma OR discrimination OR racial bias OR psychosocial stressors OR non-compliance OR financial challenge) AND (health care accessibility OR health care utilization OR patient access OR quality of assistance OR patient advocacy OR quality of care OR health services) AND (sickle cell disease OR sickle cell anemia OR Hemoglobin S disease) AND united states
