# Supplementary material for: Barriers and facilitators to healthcare utilization amongst people living with sickle cell disease in the United States: A scoping review
Source: PLoS One. 2026 Jul 6;21(7):e0349441. doi: 10.1371/journal.pone.0349441 (PMC13336462; doi:10.1371/journal.pone.0349441)
Supplement: S1 Table — (DOCX) [file pone.0349441.s003.docx]

**S1 Table: Barriers to SCD Management Reported by Studies**

| ***Author, Year*** | ***Barriers to SCD Management*** |
| --- | --- |
| Alberts 2020 [94] | Challenges of patient-centered guidelines, adherence challenges, technological barriers |
| Basu 2024 [67] | High upfront administration costs, caregiver burden |
| Baumann 2023 [77] | Technological difficulties, protocol (complex, strict), stereotypes regarding SCD, Covid-19 pandemic |
| Bediako 2016 [27] | Stigma, discrimination |
| Benjamin 2000 [38] | Bias, racism |
| Bergman 2013 [50] | Mistrust and perceived addiction political, historical, cultural, medical, and psychological dimensions |
| Blakey 2023 [28] | racism, inadequate disease knowledge, stigma, and bias |
| Boulet 2010 [39] | Racism, socioeconomic conditions, administrative issues |
| Caldwell 2019 [31] | Lack of health literacy |
| Calhoun 2022 [95] | lack of adult specialty services, stigma, operational issues, socio-economic issues |
| Carroll 2009 [30] | Poor patient-physician relationships, stigma, stereotyping |
| Chestnut 1994 [37] | Transportation problems, economic problems, daycare problems, racial discrimination |
| Crego 2020 [51] | Age-related differences in SCD |
| Crego 2021 [29] | Stigma, ED provider knowledge gaps, delays in treatment initiation and assessment |
| Crosby 2009 [5] | Poor patient-provider relationships, adverse clinic experiences, barriers to clinic attendance |
| Desai 2020 [81] | Socio Economic issues |
| Freiermuth 2014 [49] | Poor patient-physician relationships |
| Goshua 2023 [68] | High costs |
| Hankins 2012 [73] | Operational issues |
| Haque 2000 [41] | Socioeconomic status, race, place of residence |
| Hardy 2023 [83] | Poor attention (difficult to implement non-pharmacologic strategies), medical nonadherence, psychological factors |
| Haywood 2011 [45] | Poor perception regarding HU |
| Haywood 2014 [44] | Discrimination perceived by patients, lack of trust, challenges of patient-centered guidelines |
| Holdford 2021 [63] | Indirect socio-economic effects |
| Jacob 2023 [71] | Accessing SCD care, with other household responsibilities, childcare, lost time from work and school. |
| Jonassaint 2016 [46] | Lack of Knowledge, Socioeconomic Status, Disease Severity, Psychosocial Factors |
| Kam 2008 [58] | Discrimination, lack of parental decisions about their child‚ poor perception of quality of hospital care |
| Kanter 2020 [48] | Lack of empathy from clinicians, limited knowledge, provider level barrier |
| Karras 2007 [59] | Reduction in medical services, transportation, dissatisfaction with management/medical staff, issues acquiring medication |
| Kato-Lin 2014 [76] | Poor perception towards technology |
| Kirsch 2021 [78] | COVID-19 pandemic |
| Lanzkron 2008 [43] | Concerns when choosing to prescribe hydroxyurea |
| Lattimer 2010 [40] | Racism |
| Linton 2020 [52] | Health insurance, bias, concerns regarding the opioid epidemic |
| Loo 2021 [97] | Food insecurity, heat shut-off, other barriers at the family, clinic and societal levels |
| Mainous 2015 [53] | Poor patient-physician relationship |
| Masese 2019 [32] | Barriers to care, administrative issues, poor communication, and stigma. |
| Mayo-Gamble 2020 [72] | Organizational challenges, community worker challenges, and patient-driven challenges |
| Mougianis 2020 [42] | Racism, lack of social support, depression |
| Mupfudze 2021 [107] | Restrictive Medicaid eligibility criteria, low Medicaid reimbursement rates, access to transplant, issues on the carrier/care-giver level, and financial barriers |
| Nwogu-Onyemkpa 2022 [33] | Stigma, racism, gender bias, mistrust of healthcare system, misunderstanding of treatment options, preference for more aggressive care |
| Panepinto 2012 [80] | Lack of insurance |
| Payne 2007 [69] | High costs, poor adherence |
| Pecker 2023 [90] | Low educational attainment, un-familiarity with technology |
| Perry Caldwell 2021 [93] | Low health literacy levels |
| Peterson 2020 [70] | High hospital costs |
| Phillips 2022 [47] | Individual-level barriers, Family/interpersonal level barriers, provider level barriers, socio-environmental/organizational level barriers, administrative barriers |
| Power-Hays 2020 [92] | Food insecurity, difficulty paying utilities |
| Ratanawongsa 2009 [54] | Negative attitudes toward patients |
| Schlenz 2022 [60] | Patient-centered issues, Organizational issues, Logistical Challenges, Lack of Social Resources, Transfusion-Specific Resource |
| Shah 2019 [86] | High healthcare costs |
| Shelley 1994 [34] | Administrative issues, stigma, negative attitudes of physicians |
| Simmons 2019 [99] | Transportation, disability, and childcare |
| Smeltzer 2021 [55] | Provider and administrative-level barriers |
| Tanabe 2007 [74] | Gender disparities, administrative issues |
| Telfair 2003 [65] | Socioeconomic factors, location from clinic |
| Udeze, 2023 [66] | High costs |
| Utrankar 2018 [56] | Challenges of patient-centered guidelines, technological barriers, Socioeconomic barriers, mistrust of healthcare providers, and concerns regarding treatment |
| Wakefield 2018 [61] | Perceived racial bias |
| Wesley 2016 [35] | Stigma Associated with Sickle Cell Disease in Adolescents |
| Wilkie 2010 [57] | Negative beliefs regarding pain medications |
| Williams-Gray 2015 [36] | Administrative issues, racial issues, stigma, difficulties accessing knowledgeable and compassionate health care professionals |
