# Supplementary material for: Barriers and facilitators to healthcare utilization amongst people living with sickle cell disease in the United States: A scoping review
Source: PLoS One. 2026 Jul 6;21(7):e0349441. doi: 10.1371/journal.pone.0349441 (PMC13336462; doi:10.1371/journal.pone.0349441)
Supplement: S2 Table — (DOCX) [file pone.0349441.s004.docx]

**S2 Table: Facilitators to SCD Management Reported By Studies**

| ***Author, Year*** | ***Facilitators*** |
| --- | --- |
| Alberts 2020 [94] | Reminders, education, communication, autonomy |
| Bediako 2011 [82] | Positive religious coping |
| Benjamin 2000 [38] | Dedicated DH |
| Calhoun 2022 [95] | Health education efforts, autonomy, readiness, advocacy from providers, building patient-provider relationships |
| Carroll 2009 [30] | SCT-related complications |
| Crego 2021 [29] | Positive provider relationships, IPPs, EMR, coordinated care |
| Crosby 2009 [5] | Memory aids, education, positive patient-provider relationship, family support, |
| Edwards 2001 [100] | SCD-related complications, low levels of self-efficacy |
| Hankins 2012 [73] | Sense of autonomy, education, reminders, coordinated care |
| Jonassaint 2016 [46] | Medical coverage, SCD-related comorbidities |
| Karras 2007 [59] | Medicaid |
| Kato-Lin 2014 [76] | IPPs |
| Lattimer 2010 [40] | Improving patient experience, autonomy, patient-centered physician relationships |
| Loo 2021 [97] | Close clinical-provider relationships |
| Masese 2019 [32] | IPPs, comfort prescribing opioids, electronic medical records |
| Mayo-Gamble 2020 [72] | Community level strategies |
| Pecker 2023 [90] | Telemedicine |
| Peterson 2020 [70] | SCT-related complications |
| Ratanawongsa 2009 [54] | Education |
| Schlenz 2022 [60] | Positive experiences, positive staff relationships, access to blood bank, SES help |
| Shelley 1994 [34] | Education, community aspect, autonomy |
| Simmons 2019 [99] | Community aspect, reminders, remote delivery |
| Simpson 2017 [88] | Dedicated DH |
| Telfair 2003 [65] | SCD related complications, SES status |
| Utrankar 2018 [56] | Education, autonomy, rewards for guideline adherence, technology |
