# Supplementary material for: Barriers and facilitators to healthcare utilization amongst people living with sickle cell disease in the United States: A scoping review
Source: PLoS One. 2026 Jul 6;21(7):e0349441. doi: 10.1371/journal.pone.0349441 (PMC13336462; doi:10.1371/journal.pone.0349441)
Supplement: S3 Table — (DOCX) [file pone.0349441.s005.docx]

**S3 Table: Healthcare Access/Utilization Reported by Studies**

| ***Author, Year*** | ***Healthcare Utilization*** |
| --- | --- |
| Barriteau 2023 [64] | Hospital admissions |
| Basu 2024 [67] | Gene therapy |
| Baumann 2023 [77] | ED/hospital utilization |
| Bediako 2011 [82] | Hospital admissions |
| Benjamin 2000 [38] | DH (day-hospital) visits |
| Blakey 2023 [28] | Physician visits |
| Boulet 2010 [39] | Medical specialist, ED visits |
| Caldwell 2019 [31] | Healthcare encounters, clinic visits, ED utilization and hospitalizations |
| Carroll 2009 [30] | Inpatient admissions and outpatient services (i.e. office visits, hematologist, infusion clinic, OBGYN) |
| Chestnut 1994 [37] | Adherence to physician recommendations |
| Crego 2020 [51] | Hospitalization and outpatient services |
| Crego 2021 [29] | ED visits |
| Crosby 2009 [5] | Self-reported adherence to clinic appointments |
| Desai 2020 [81] | Inpatient hospitalization and outpatient services |
| Goshua 2023 [68] | Gene therapy |
| Hankins 2012 [73] | Clinic visits and outpatient services, large gaps between leaving the pediatric program and initiating adult care |
| Haque 2000 [41] | Clinic visits |
| Hardy 2023 [83] | ED visits, hospitalization |
| Haywood 2011 [45] | Hydroxyurea (HU) therapy |
| Jacob 2023 [71] | Physician visits, Telemedicine |
| Jonassaint 2016 [46] | ED utilization |
| Kam 2008 [58] | Hospital admissions |
| Kanter 2020 [48] | ED visits, hospital admissions |
| Karras 2007 [59] | Hospital and clinic visits |
| Kato-Lin 2014 [76] | Digitization of paper-based individualized pain plans |
| Kirsch 2021 [78] | Emergency Department utilization |
| Linton 2020 [52] | Emergency department visits |
| Mainous 2015 [53] | Physician utility of CDS tools |
| Mayo-Gamble 2020 [72] | Clinic Visits, Health Fairs |
| Molokie 2018 [85] | Opioid use, ER visits, Acute Care Units |
| Mupfudze 2021 [107] | Allo HSCT/Bone Marrow Transport (BMT) |
| Nwogu-Onyemkpa 2022 [33] | Palliative care services, hospitalizations |
| Panepinto 2012 [80] | Hospital visits, acute care |
| Payne 2007 [69] | Iron Chelation Therapy |
| Pecker 2023 [90] | Telemedicine |
| Peterson 2020 [70] | Inpatient hospitalizations, ED visits |
| Raphael 2013 [84] | ED encounters, hospital admissions |
| Schlenz 2022 [60] | Chronic Red Cell Transfusion Therapy |
| Shah 2019 [86] | Patient hospital visits, outpatient office visits, outpatient ER visits, and in-patient visits |
| Shankar 2008 [87] | Medical care utilization (outpatient/ED visits) |
| Simmons 2019 [99] | Non-pharmacologic therapy and non-opioid pharmacologic therapy |
| Simpson 2017 [88] | ED visits, ED length of stay (LOS) |
| Tanabe 2007 [74] | ED Visits |
| Telfair 2003 [65] | Clinical services |
| Udeze, 2023 [66] | Inpatient admissions, ED visits |
| Wilkie 2010 [57] | Emergency Department (ED) visits or hospitalization |
| Zhang 2021 [108] | Opioids therapy |
