# Supplementary material for: Barriers and facilitators to healthcare utilization amongst people living with sickle cell disease in the United States: A scoping review
Source: PLoS One. 2026 Jul 6;21(7):e0349441. doi: 10.1371/journal.pone.0349441 (PMC13336462; doi:10.1371/journal.pone.0349441)
Supplement: S4 Table — (DOCX) [file pone.0349441.s006.docx]

**S4 Table: EBIs Reported by Studies**

| ***Author, Year*** | ***Control Condition*** | ***Experimental Condition/Intervention*** | ***Intervention Duration*** | ***Main Findings*** |
| --- | --- | --- | --- | --- |
| Baumann 2023 [77] | N/A | Implementing NHLBI recommendations for vaso-occlusive pain events (VOE) treatment by embedding Individualized Pain Plans in the electronic health record (E-IPP) | February to March of 2022 | Findings from this study contribute to learning how to implement E-IPPs for adult patients with SCD in ED. The study findings highlight the importance of early engagement with different team members, a champion from the emergency department, study coordinators with different skills and enhancement of communication and trust among team members |
| Benjamin 2000 [38] | N/A | The specific procedures in the DH are listed below, (1)Assess pain; (2)Select drug and loading doses, (3)Titrate medication to relief; (4)Use by the clock dosing to maintain relief; (5)Adjust rescue dosing for breakthrough pain; (6)Combine drugs to enhance the efficacy/toxicity ratio; (7)Adjust drugs for tolerance; (8)Evaluate, record, and treat adverse events; (9)Adjust method and route of drug delivery; (10)Identify and treat precipitating factors; (11)Making depositions based upon response to therapy and the presence or absence of comorbidities | 5 years | We conclude that a dedicated facility provides the kingpin for effective and rapid painful crisis management, reduces hospitalizations, and facilitates integration of the approach into other areas of care. |
| Hankins 2012 [73] | Adolescents who chose not to participate were given information about the available adult SCD programs in the region, and the pediatric hematology nurse case manager offered to make their first appointment | The Program has three components: a tour of adult SCD programs, a lunch discussion with the pediatric staff (immediately following the tour), and scheduling of the first visit to the adult SCD program by the pediatric hematology nurse case manager | The tour was conducted 4x/year (approximately once every 3 months) from June 2007 to December 2008 | This transition pilot program was feasible, and most adolescent participants with SCD established an adult medical home |
| Kato-Lin 2014 [76] | N/A | In May 2010, these paper-based pain management plans were scanned into Cerner MilleniumTM EHR, allowing all clinical professionals to access the personalized  pain plans at any time in any location. The roll-out of the digitization was followed by the elimination of the binder containing paper plans from the ED. | N/A | This study highlights the important role of health information technology (HIT) on vaso-occlusive pain management for pediatric patients with sickle cell disease and the critical challenges in accommodating human factor considerations in implementing and evaluating HIT effects |
| Mayo-Gamble 2020 [72] | N/A | Strategies leveraged to engage sickle cell disease patient partners were designed to be accomplished through employment of CHAs | N/A | The present study demonstrates the use of a CHA training model as an effective mechanism for recruiting and engaging the SCD community as partners in PCOR. |
| Power-Hays 2020 [92] | N/A | Paper screener followed by a referral to local community organizations for the specific needs endorsed. | N/A | There is a high burden of SDoH in families of children with SCD. Universal screening in a pediatric hematology clinic with the subsequent connection of patients with SCD to community resources is feasible using existing clinic resources |
| Simmons 2019 [99] | Wait-list control participants received usual care from the SCD clinic and had the option to take the MBI upon completion of their study participation | Those assigned to the MBI started the program, which included six weekly, 60-minute telephonic group classes led by the same certified MBI instructor with over 10 years of mindfulness instructional experience, including two years leading telephonic groups and group-based MBIs for research | 6 weeks | A MBI is feasible and acceptable for persons with SCD experiencing chronic pain |
| Simpson 2017 [88] | N/A | Ten patients (five women) had an ED-based care plan developed. We confirmed use of BPAs and the ED protocol in all patients during ED visits. | 12/31/13 - 01/01/14 | A targeted approach is both feasible and potentially effective |
