# Supplementary figures and images for: Barriers and facilitators to healthcare utilization amongst people living with sickle cell disease in the United States: A scoping review
Source: PLoS One. 2026 Jul 6;21(7):e0349441. doi: 10.1371/journal.pone.0349441 (PMC13336462; doi:10.1371/journal.pone.0349441)

**S5 Table: RoB Assessment**

**
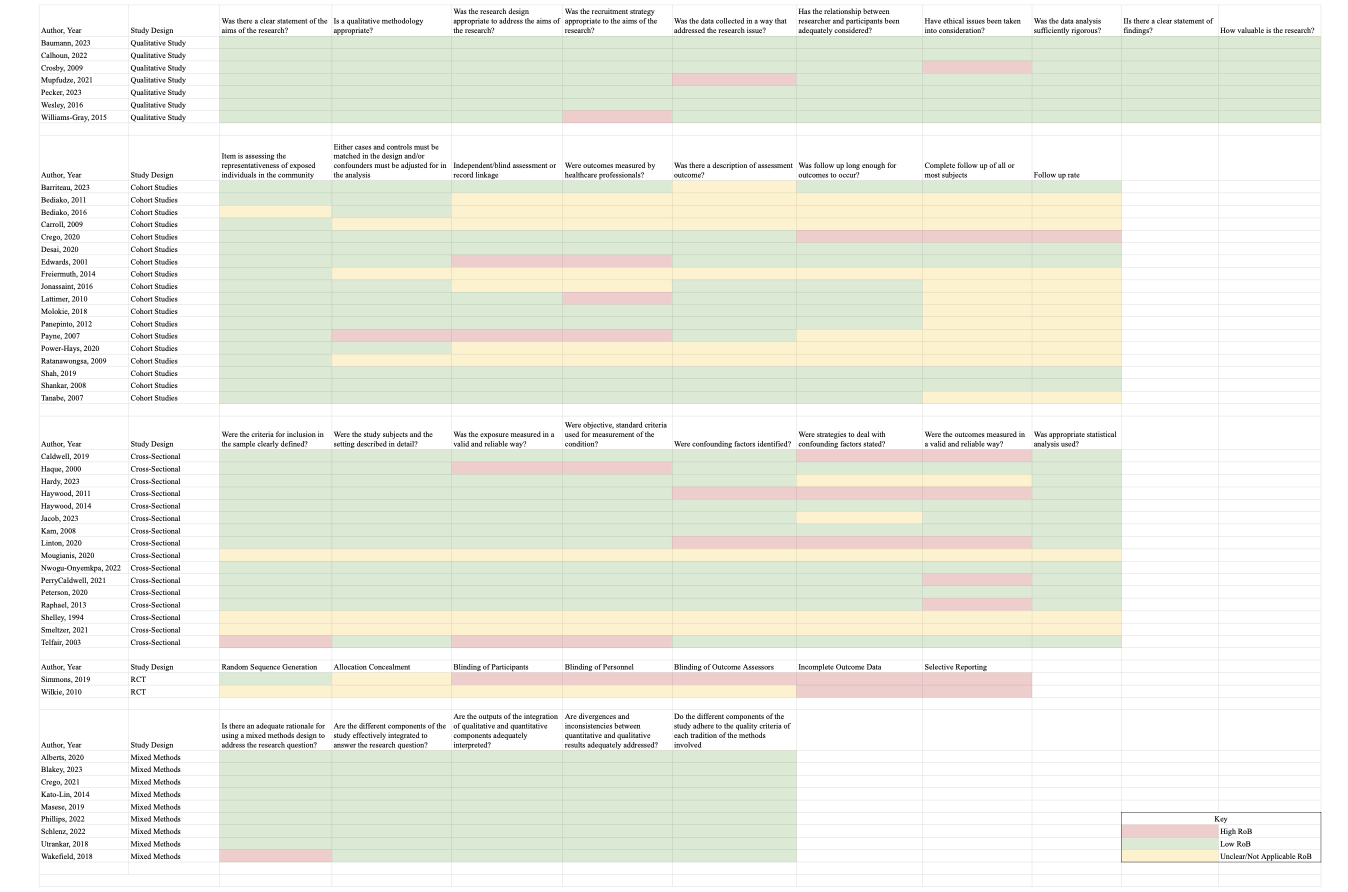
**

Supplement: S5 Table — (DOCX) [file pone.0349441.s007.docx]
